# Recent advances in ratiometric electrochemical sensors for food analysis

**DOI:** 10.1016/j.fochx.2024.101681

**Published:** 2024-07-23

**Authors:** Xincheng Hu, Wei Wei, Xinyi Li, Yewen Yang, Binbin Zhou

**Affiliations:** a College of Chemistry and Chemical Engineering, Henan Engineering Center of New Energy Battery Materials, Shangqiu Normal University, Shangqiu 476000, China; b College of Chemistry and Chemical Engineering, Hunan Institute of Science and Technology, Yueyang 414006, China; c School of Chemistry and Chemical Engineering, Hunan University of Science and Technology, Xiangtan 411201, China

**Keywords:** Ratiometric electrochemical sensors, Food analysis, Sensor design strategies

## Abstract

Ratiometric electrochemical sensors are renowned for their dual-signal processing capabilities, enabling automatic correction of background noise and interferences through built-in calibration, thus providing more accurate and reproducible measurements. This characteristic makes them highly promising for food analysis. This review comprehensively summarizes and discusses the latest advancements in ratiometric electrochemical sensors and their applications in food analysis, emphasizing their design strategies, detection capabilities, and practical uses. Initially, we explore the construction and design strategies of these sensors. We then review the detection of various food-related analytes, including nutrients, additives, metal ions, pharmaceutical and pesticide residues, biotoxins, and pathogens. The review also briefly explores the challenges faced by ratiometric electrochemical sensors in food testing and potential future directions for development. It aims to provide researchers with a clear introduction and serve as a reference for the design and application of new, efficient ratiometric electrochemical sensors in food analysis.

## Hightlights

• Ratiometric electrochemical (EC) sensor as a novel and effective analytical method.
• Comprehensive review of ratiometric EC sensors' applications in food analysis.
• Discussion on the construction principles and methods of these sensors.
• Overview of ratiometric EC sensors for diverse analytes in food analysis.

## Graphical abstract

**Figure:**
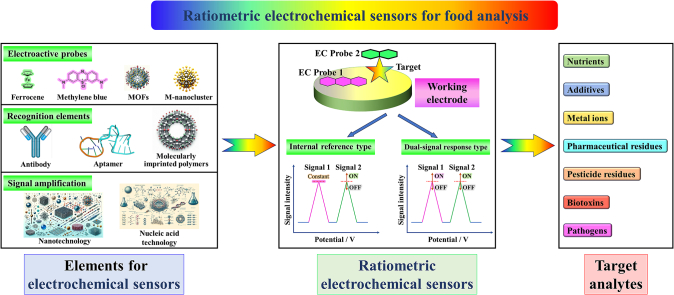

## 1 Introduction

Food analysis plays a pivotal role in ensuring food safety and quality control (Chen et al., 2020; Godfray et al., 2010). This field focuses on evaluating the nutritional value, freshness, additive usage, and potential toxic substances that may emerge during various food processing stages. The scope of food analysis is vast, encompassing a wide array of food types including vegetables, meats, fruits, dairy products, drinking water, and alcoholic beverages. Each category requires meticulous identification of components and potential hazards like toxins, pesticides, heavy metals, illegal additives, residual pharmaceuticals, as well as microbial contaminants such as bacteria and viruses. Moreover, food products are susceptible to contamination at multiple points including during production, packaging, storage, cooking, and transportation. The significance of food analysis extends beyond safeguarding consumer health and safety; it is also critical for complying with regulatory standards and facilitating market access in the global food trade. In response to these challenges, the field has evolved to develop and adopt a variety of efficient, rapid, and sensitive detection techniques. Commonly employed methods include high-performance liquid chromatography (HPLC) (Donno, Mellano, Gamba, Riondato, & Beccaro, 2020; Riboni et al., 2023; Tsiasioti & Tzanavaras, 2024), gas chromatography (Duan, Dong, Dong, & Gao, 2021; Nolvachai, Amaral, & Marriott, 2023; Peñalver, Arroyo-Manzanares, Campillo, & Viñas, 2021), colorimetric tests (Hou, Fu, Ju, & Wu, 2020; Liu & Dong, 2023; Singh, Singh, Kaur, & Singh, 2022), fluorescence spectroscopy (Duan et al., 2021; Wan, Pang, Feng, & Shen, 2022; Wen et al., 2023), Raman spectroscopy (Neng, Wang, Wang, Zhang, & Chen, 2023; Wu et al., 2023; Zhang, Ma, & Sun, 2021), and electrochemical sensing techniques (Ayerdurai, Cieplak, & Kutner, 2023; Jiang et al., 2024; Mohan, Priyanka Singh, Chauhan, Pombeiro, & Ren, 2023; Xing, Zhang, Fu, Lorenzo, & Hao, 2022; Zhou et al., 2024).

Electrochemical methods are especially effective in the field of food analysis due to their unique advantages (Gu et al., 2022; Xu, Yang, Zhang, Lu, & Bai, 2023; Zhang et al., 2021; Zhou et al., 2023). These methods are renowned for their high sensitivity, rapid response times, and minimal sample preparation requirements. Additionally, compared to traditional analytical techniques such as chromatography and mass spectrometry, electrochemical methods are generally more cost-effective and portable. Ratiometric electrochemical sensors mark a significant advancement in electrochemical analysis technologies (Kodr et al., 2021; Liu, Dong, Zhang, & Tian, 2017; Luo et al., 2022; Qin, Liu, Meng, Liu, & You, 2024; Zhang et al., 2022). Unlike traditional sensors that rely on a single signal output, ratiometric sensors employ two electrochemical signals and use the ratio of these signals as the output. This dual-signal approach provides inherent error correction capabilities, which are absent in traditional single-signal sensors, thereby greatly enhancing accuracy, sensitivity, and reliability. The primary advantage of ratiometric sensors in food analysis is their ability to correct for background noise and potential interferences that could affect measurements. This feature is particularly valuable in complex food matrices, where various substances may interfere with the detection of specific analytes. Additionally, ratiometric sensors often incorporate built-in correction features, which enhance their sensitivity and allow for more precise quantification of target analytes.

**Fig. 1:**
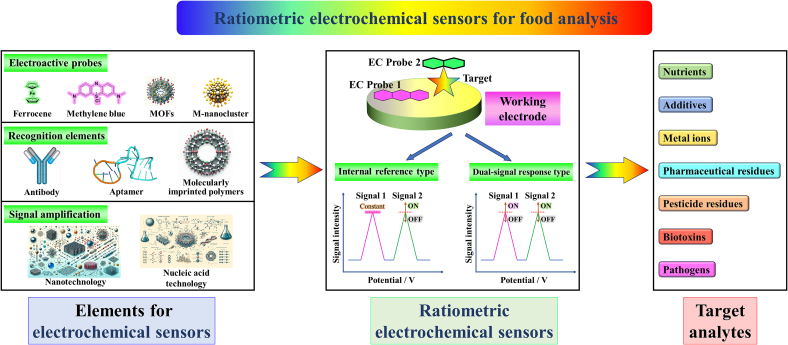
Schematic illustration of the construction of ratiometric electrochemical sensors and their application in food analysis.

To date, a few review articles have summarized and analyzed ratiometric electrochemical sensors (Cui, Li, Zou, & Zhang, 2018; Jin, Sun, Sun, & Gui, 2021; Spring, Goggins, & Frost, 2021; Wei et al., 2022; Xu, Wang, & Liu, 2022; Yang et al., 2018; Zhang, Wen, Wang, Yang, & Sun, 2022; Zhu, Wang, Yang, Chen, & Yu, 2023), contributing significantly to the field. However, reviews specifically discussing the application of ratiometric electrochemical sensors in food analysis are still relatively rare. Given the importance of food analysis and the advantages of ratiometric electrochemical sensors, this review paper will systematically and comprehensively outline the application of ratiometric electrochemical sensors in food analysis. The paper will first introduce the types of ratiometric electrochemical sensors (internal reference type and dual-signal response type) and the construction principles of those used in food analysis. We will then detail the detection of various targets in food analysis, including the construction, detection principles, and analytical performance of the sensors. Finally, this review will provide insights into the current state and future development of ratiometric electrochemical sensors in food analysis. We believe this review will offer researchers in the field a clear introduction to the application of ratiometric electrochemical sensors in food analysis and guide the design and application of new, efficient ratiometric electrochemical sensors (Fig. 1). (See Table 1.)

**Table 1:** A summary of the ratiometric electrochemical sensors for food analysis.

**Analyte**	**Working electrode**	**Ratiometric** **Signal probe**	**Linear range**	**LOD**	**Real samples**	**Reference**
**Nutrients**						
Quercetin	GCE	Quercetin/TH	0.1–15 μM	3.1 nM	Juice, Tea	(Yu et al., 2017)
Caffeic acid	PMB@Ni–TPA/GCE	CEA/pMB	0.25–15 μM	0.20 μM	Tea	(Yin, et al., 2021b)
Propyl gallate	AQ /MWCNT/CS/GCE	PG/AQ or PG/MWCNT	1–30 μM	0.67 μM	Oil	(Yin, et al., 2021a)

**Additives**						
Nitrite	AgNC@NCS SPE	NO_2_^−^/AgNC	1.12–1400 μM	0.38 μM	Vegetable, Milk	(Yin et al., 2022)
Nitrite	SPE	NO_2_^−^/OPD	10–300 μM	4.7 μM	Pickle water	(Niu et al., 2022)
Nitrite	SPE	NO_2_^−^/TMB	3–250 μM	1.7 μM	Egg	(Wang et al., 2022)
Nitrite	Cu-MOFs/EGP	NO_2_^−^/TMB	0.62–200 μM	0.57 μM	Sausage, Eggs, vegetables	(Pan et al., 2023)
Malachite green	Cu − MOF/CPE	MG/Cu_3_(HHTP)_2_	5–1000 nM	1.34 nM	Fish	(Li, et al., 2023a)
TBHQ	MnO_2_/ERGO/GC	TBHQ/MnO_2_	1.0–50.0 μM; 100.0–300.0 μM	0.8 μM	Oil	(Cao et al., 2019)
TBHQ	Co NC/CNT/MB/GCE	TBHQ/MB	2–100 μM	0.054 μM	Oil	(Zhang, et al., 2024b)
Vanillin	aptamer-AuNPs/Fc-KB/ZIF-8@GCE	Vanillin/Fc	10 nM–0.2 mM	3 nM	Chocolate, Nougat	(Sun et al., 2019)
Kojic acid	MXene/PB/AuNPs/GCE	KA/PB	1–600 μM	1 μM	Apple cider vinegar	(Karuppaiah et al., 2023)
Glutathione	Ag/NiFe PBA/GCE	Ag/Fe	0.5 nM – 5 μM	0.12 nM	Cherry tomato, cucumber, grape	(Xu, et al., 2022b)
Sunset yellow	PDA/NiS@HCS/GCE	SY/PDA	0.01–100 μM	3 nM	Vinegar,Wine	(Chen et al., 2022)
Sunset yellow	MIPs-ir-AuNPs/ITO	SY/AuNPs	10 nM– 100 μM	1.6 nM	Orange juice	(Wu, et al., 2023b)
Bisphenol A	PTB/AuNP/GCE	BPA/PTB	0.2–5 μM	0.15 μM	Water sample	(Sun et al., 2021)
Bisphenol A	COF/AgNPs/GCE	BPA/AgNPs	0.5–100 μM	0.15 μM	Tea, Juice, Beer	(Pang et al., 2022)
H_2_O_2_	AuNPs/PTH/KB/GCE	4-AP/PTH	0.3–100 μM	0.26 μM	Milk, Beer, Juice	(Luo, et al., 2022a)
H_2_O_2_	MB@ZIF-8/COOH-MWCNTs/GCE	4-AP/MB	5–200 μM	0.2 μM	Milk	(Zhao et al., 2023)

**Metal ions**						
Cd^2+^	ssDNA@PTh-Au/Apt/AuNPs/SPE	Cd^2+^/PTh-Au	2 × 10^−3^–2.2 × 10^−3^ mg∙L^−1^	0.7 ng∙L^−1^	Mussel	(Chen et al., 2021)
Cd^2+^; S.aureus	ZrO_2_/Ni/Co-MOFs@Au NPs/Fc-S1/MCH/AQ-S2/MB-S3	MB/Fc AQ/Fc	1 × 10^−2^–1 × 10^4^ ng∙L^−1^; 1 × 10^1^–1 × 10^7^ CFU∙mL^−1^	0.191 pg∙L^−1^; 1.37 CFU∙mL^−1^	Scallop, Fish	(Wang, et al., 2023b)
Pd^2+^	S1/AgNWS@ZIF-8/MB/MCH/Apt/AuNPs/GCE	Fc/MB	20 nM–9 μM	10 nM	Fish	(Han et al., 2023)
Cd^2+^, pd^2+^	p-CCM/MWCNTs/LDHs/GCE	Cd^2+^/p-CCM; Pd^2+^/p-CCM	10–500 μg∙mL^−1^; 20–1000 μg∙mL^−1^	0.61 μg∙mL^−1^; 0.74 μg∙mL^−1^	Rice flour, Wheat flour	(Duan et al., 2024)

**Pharmaceutical and pesticide residues**						
Tetracycline	BSA/Fc-Apt/AuNPs-CS/SPCE; BSA/Apt/AuNPs/CNFs/SPCE	CNFs/Fc	10^−8^–10^−3^ g∙L^−1^	3.3 × 10^−7^ g∙L^−1^	Milk	(Xu et al., 2017)
Carbendazim	MXene@AgNCs/NH_2_-MWCNTs/GCE	CBZ/AgNCs	0.3 nM −10 μM	0.1 nM	Lettuces	(Zhong et al., 2021)
Kanamycin	Fc-HP-polyA@AuNPs/GDY-MB/ATO/AuE	Fc/MB	0.05–1 μM	6.044 nM	Milk	(Gao et al., 2022)
Kanamycin	Apt/AuNPs/GCE	Fc/MB	1 × 10^−4^–10^2^ ng∙mL^−1^	74 fg∙mL^−1^	Milk, Honey	(Jin et al., 2024)
Trimethoprim	MIP-MoS_2_@CNTs/GCE	TMP/Fc	0.05–3 μM	27.01 nM	Fish	(Deng et al., 2024)
Phoxim	BSA/m-Ab/MB/DTNS/AuNPs/GCE	Phoxim/MB	0.1–30 μg∙L^−1^	3 ng∙L^−1^	Vegetables	(Su, et al., 2022a)
Parathion-methyl	MXene-Au-ATG/PTM/BSA/Ab/AuNPs/SPE	PTM/MB	0.02–38 ng∙mL^−1^	0.01 ng∙mL^−1^	Cabbage	(Su, et al., 2022b)
Malathion; Profenofos	BSA/Pb^2+^-APT_1_&Cd^2+^-APT_2_/HP-TDN_TH_/Au@ZIF-8/AuE	Pb/TH; Cd/TH	5–5 × 10^4^ pg∙mL^−1^; 50–5 × 10^4^ pg∙mL^−1^	4.3 pg∙mL^−1^; 13.3 pg∙mL^−1^	Lettuce, Spinach	(Li, et al., 2023b)
Imidacloprid	MIP/FcHT/AuNPs/GCE	IMI/FcHT	0.5–100 μM	47 nM	Fruits	(Zhang, et al., 2020b)
Imidacloprid	β-CD/PB/AuNPs/GCE	IMI/PB	0.2–120 μM	61 nM	Fruits	(Liu et al., 2021)
Carbaryl	GCE	carbaryl/Nile blue	5–75 μM	1 μM	Tomato, Cabbage	(Zhang, et al., 2020a)
Glyphosate	CNTs-Cu MOFs/MB/GCE	Cu/MB	0.5–400 nM	0.014 nM	Potato	(Zhao et al., 2024)

**Biotoxins**						
Aflatoxin B1	MB-cDNA/Fc-P/AuE	Fc/MB	1 pM – 50 nM	0.43 pM	Peanut, Maize, Wheat	(Wu et al., 2017)
Aflatoxin B1	Bi/GCE	Pd/Cd	5 × 10^−3^– 50 ng∙mL^−1^	4.5 pg∙mL^−1^	Peanut	(Wang et al., 2019)
Aflatoxin B1	BSA/Ab1/MWCNTs/Fc-MOF/GCE	BPYHBF_(ECL)_ /Fc_(EC)_	10^−5^– 100 ng∙mL^−1^	5.3 fg∙mL^−1^	Walnut	(Lv et al., 2022)
Aflatoxin B1	Aptamer/AuNFs-cDNA/ITO	MB/Fc	0.1–1000 pg∙mL^−1^	0.032 pg∙mL^−1^	Peanut	(Cui et al., 2022)
Aflatoxin B1	GCE	MB/Fc	0.1–100 ng∙mL^−1^	38.8 pg∙mL^−1^	Corn, Wheat, Peanut, Rice	(Zhu et al., 2022)
Aflatoxin B2	Fc-Apt-Fc/CS/TH-rGO/GCE	TH/Fc	0.001–10 ng∙mL^−1^	0.19 pg∙mL^−1^	Peanut	(Jia et al., 2022)
Aflatoxin B1	TH-rGO/CS/Apt-cDNA/ GCE	TH/Fc	10^−3^–10^3^ ng∙mL^−1^	0.33 pg∙mL^−1^	Peanut	(Lv et al., 2023)
Aflatoxin B1	Apt-Fc/MCH/ssDNA-MB/Au NPs/PC_ZIF-8_-ZnO/WE	Fc/MB	10^−4^–10^2^ ng∙mL^−1^	36.7 fg∙mL^−1^	Peanuts, Corn	(Zhang, et al., 2024a)
Aflatoxin B1	Fc-S1-H4/MCH/AuNPs/GCE	Fc/MB	10^−4^–10 ng∙mL^−1^	61 fg∙mL^−1^	Flour, Soybean	(Liang et al., 2024)
Ochratoxin A	MCH/sDNA/MB-rp/APT/GE	Ru(phen)_3_^2+^_(ECL)_/ MB_(EC)_	32.30 pM–12.40 nM	17.30 pM	Corn, Moldy Oat	(Lin et al., 2018)
Ochratoxin A	hDNA/Apt/cDNA/AuE	Fc/MB	10^−2^–10 ng∙mL^−1^	3.3 pg∙mL^−1^	wheat	(Zhu et al., 2020)
Ochratoxins	Antigen/CdS/MoS_2_ NFs/FTO	CdS/MoS_2(PEC)_/ Zn_(EC)_	1 ng∙L^−1^ –1 μg∙L^−1^	0.1 ng∙L^−1^ (OTA) 0.5 ng∙L^−1^ (OTB) 0.5 ng∙L^−1^ (OTC)	Millet, Maize	(Qileng, et al., 2020b)
Ochratoxin A	HP-Fc/AuE	MB/Fc	0.005–50 ng∙mL^−1^	2.35 pg∙mL^−1^	Corn	(Liang et al., 2021)
Ochratoxin A	MIP/Au NPs/PIL-FMNS/CNT-MoS_2_/GCE	OTA/FMNS	0.5 μM −15 μM	14 nM	Wines	(Hu et al., 2022)
Ochratoxin A	OTA Apt/MCH/DNA NTH/Au NPs@MXene/GCE	Au NPs@UiO-66/ [Fe(CN)_6_]^3−/4−^	1 pg∙mL^−1^ –100 ng∙mL^−1^	330 fg∙mL^−1^	Corn	(Li, et al., 2022b)
Ochratoxin A	DNA2-AuNPs@Co-MOFs/TDN/Au NPs@Ti_3_C_2_ MXene/GCE	TB/Co-MOFs	1–50 ng∙mL^−1^	0.31 fg∙mL^−1^	Wines	(Guan et al., 2023)
Ochratoxin A	H1/AuNPs/GCE	MB/Fc	10^−4^–50 ng∙mL^−1^	81 fg∙mL^−1^	Flour, Barley, Oats, Maize	(Liu, et al., 2023c)
Ochratoxin A	AuE/NPG/MB-Apt/MCH/Fc-cDNA	MB/Fc	1 pg∙mL^−1^ –100 ng∙mL^−1^	0.31 fg∙mL^−1^	Cordyceps sinensis, Grape juice	(Wang et al., 2024)
Ochratoxin A Aflatoxin B1 Zearalenone	Antigen/CMGC film + CuS@Ab_2_	CMGC_(PEC)_/CuS_(EC)_	0.001–1 μg∙L^−1^	AFB1 0.17 ng∙L^−1^ OTA 0.59 ng∙L^−1^ ZEN 0.60 ng∙L^−1^	Maize, Millet	(Qileng, et al., 2020a)
Zearalenone	DNA/AuE + MB-Au@Pt/Fe-N-C-dsDNA	MB/Ag	10^−5^–10 ng∙mL^−1^	5 fg∙mL^−1^	Flour	(Suo et al., 2022)
Patulin	Fc-apt/CdTe QDs/Au NRs/GCE	CdTe _(PEC)_/Fc_(EC)_	5 × 10^−5^–500 ng∙mL^−1^	30 fg∙mL^−1^	Apple	(Liu, et al., 2023b)
Deoxynivalenol	DON-BAS/AuE	Fe-MOF/ [Fe(CN)_6_]^3−/4−^	0.5–5000 pg∙mL^−1^	0.0166 pg∙mL^−1^	Flour	(Shu et al., 2024)
Citrinin	MIP/[APMIm]Br/BN-HPC/GCE	[Fe(CN)_6_]^3−/4−^/TH	0.001–10 ng∙mL^−1^	0.1 pg∙mL^−1^	Rice, Wheat	(Hu et al., 2021)
Saxitoxin	GCE	AgCl/Fe[Fe(CN)_6_]^−^	0.04–0.15 μM	1 nM	Clams Shrimps	(Zeng et al., 2024)
Streptomycin	GCE/CP/Apt/MCH	MB/Fc	0.1 pM – 10 nM	0.008 pM	Milk, Honey	(Zhang et al., 2023)
Microcystin	MB-ssDNA/MCH/SH-ssDNA/AuE	Fc/MB	0.005–2.5 nM	1.5 pM	Fish	(Li, et al., 2022a)
Cry1ab protein	ITO/CdTe QDs/Au NRs/Ab_1_/BSA	CdTe@CdSe_(PEC)_/ (CdTe@CdSe+MB)_(PEC)_	0.01–100 ng∙mL^−1^	1.4 pg∙mL^−1^	Corn, Wheat	(Meng et al., 2022)

**Pathogens**						
Salmonella	GCE	Fc-based probes	1.03 × 10^5^–1.1 × 10^10^ CFU∙mL^−1^ (Salmonella esterase)	39.27 × 10^3^ CFU∙mL^−1^	Milk	(Kumaravel et al., 2022)
Salmonella	GQDs-AuNPs/GCE	Fc-based probes	10^3^–10^10^ CFU∙mL^−1^ (Salmonella esterase)	356.2 CFU∙mL^−1^	Blood, Milk	(Kumaragurubaran et al., 2023)
Salmonella	SH-β-CD/AuNPs/GCE	MB/Fc	3 × 10^−5^–30 ng∙μL^−1^	15.8 fg∙μL^−1^	Meat, Milk, Egg	(Yu et al., 2022)
Salmonella	Fc-hp/AuNPs/GCE	Fc/MB	5.8 × 10^−6^–5.8 ng∙μL^−1^	2.08 fg∙μL^−1^	Meat, Milk, Egg, Cheese	(Zheng et al., 2023)
*S. aureus*	1-MB/MXene@Au NPs/GCE	Ru_(ECL)_/MB_(EC)_	5–10^8^ CFU∙mL^−1^	1 CFU∙mL^−1^	Chicken, Spinach, Milk	(Shan, et al., 2023a)
*S. aureus*	H1/Au NPs@ZIF-MOF/GCE	MB/Fc	5–10^8^ CFU∙mL^−1^	1 CFU∙mL^−1^	Juice, Milk	(Shan, et al., 2023b)

Abbreviations: [APMIm]Br, 1-Aminopropyl-3-methylimidazolium bromide. 4-AP, 4-Aminophenol. ABAPE, 4-Aminophenylboronic acid pinacol ester. AgNC, Silver nanoclusters. Apt, Aptamer. AQ, Anthraquinone. ATG, Antigen for parathion-methyl. ATO, Antimony tin oxide. AuE, Gold electrode. AuNPs, Gold nanoparticles. BN-HPC, Boron and nitrogen co-doped hierarchical porous carbon. BPA, Bisphenol A. BSA, Bovine serum albumin. CBZ, Carbendazim. CEA, Caffeic acid. CMGC, CdS nanoparticles/MoS2 nanoflakes/reduced graphene oxide/carbon nanotubes. CNFs, carbon nanofibers. CNT, carbon nanotube. Co NC/CNT, carbon nanotube-encapsulated Co/nitrogen-doped carbon. Co NC, Co/nitrogen-doped carbon. CPE, Carbon paste electrode. CS, Chitosan. DON, Deoxynivalenol. DTNS, DNA tetrahedron nanostructure. EGP, Exfoliated graphite paper. ERGO, Electrochemically reduced graphene oxide. Fc, Ferrocene. FcHT, 6-(Ferrocenyl)hexanethiol. FMNS, Flavin mononucleotide. GCE, Glassy carbon electrode. GDY, Graphdiyne. HCS, Hollow carbon spheres. HHTP, 2,3,6,7,10,11-Hexahydroxytriphenylene. HP-TDN, Hairpin tetrahedral DNA nanostructure. IMI, Imidacloprid. ITO, Indium‑tin-oxide. KA, kojic acid. KAN, Kanamycin. KB, Ketjen black. LDHs, Layered double hydroxides. MAL, Malathion. MB, Methylene blue. MCH, 6-Mercapto-1-hexanol. MG, Malachite Green. MIPs, Molecularly imprinted polymers. MWCNT, Multi-walled carbon nanotube. NCS, Nitrogen-doped carbon. NiFe PBA, Nickel and iron contained Prussian Blue analog. NPG, Nanoporous gold. OPD, o-Phenylenediamine. PABA, *p*-Aminophenylboronic acid. PB, Prussian blue. p-CCM, Poly-curcumin. PC_ZIF-8_, ZIF-8-derived porous carbon. PDA, Poly-dopamine. PG, Propyl gallate. PMB, Poly(methylene blue). PRO, Profenofos. PTB, Poly(toluidine blue). PTH, Poly(thionine). PTM, Parathion-methyl. SPCE, Screen printed carbon electrode. SPE, Screen-printed electrode. *S. aureus*, *Staphylococcus aureus*. SY, Sunset Yellow. TBHQ, Tert-Butylhydroquinone. TDN, Tetrahedral DNA nanostructure. TET, Tetracycline. TH, Thionine. TMB, 3,3′,5,5’-Tetramethylbenzidine. TPA, Terephthalic acid. TMP, Trimethoprim. WE, Working electrode. β-CD, β-Cyclodextrin.

## 2 Design strategies for ratiometric electrochemical sensors

Ratiometric electrochemical sensors utilize the ratio of signals from multiple redox-active molecules to provide signal output. These advanced devices are distinguished by their ability to correct for background noise, which offers more reliable and sensitive measurements than those provided by traditional electrochemical sensors. It is important to note that ratiometric electrochemical sensors differ from conventional dual-signal electrochemical biosensors. Conventional dual-signal biosensors use two independent signal sources for qualitative and quantitative analysis of the target, enhancing detection reliability. In contrast, ratiometric electrochemical sensors generally use the ratio of two signals to correct background noise and interference, thereby improving detection accuracy and repeatability. A primary advantage of ratiometric sensors is their “built-in calibration” feature, typically arising from the use of two electrochemically active probes that respond to environmental changes under similar conditions. This setup helps counteract variable external factors, ensuring more accurate and consistent readings. The characteristics and corresponding design approaches of ratiometric electrochemical sensors mainly fall into two categories (Fig. 1).

### 2.1 Internal reference type

In this design, a fixed internal reference signal (I) is established, which remains unchanged upon the introduction of the target analyte. Another signal (II) that varies with the concentration of the target analyte is produced. The target is analyzed accurately using the response ratio (S_I_/S_II_). The electroactive probes generating signals are diverse, including electroactive small molecules (such as ferrocene, methylene blue, toluidine blue, thionine, Nile blue), metal-organic frameworks (MOFs), metallic nanoclusters, oxide nanomaterials, etc. The generation of the electrochemical signal (II) can be of two types: direct electrochemical signals produced by the oxidation/reduction of the target analyte itself, and indirect electrochemical signals often achieved through antigen-antibody recognition, nucleic acid aptamer binding, followed by the introduction of additional signal probes.

### 2.2 Dual-signal response type

This strategy employs two different electrochemical probes, each responsive to the target analyte, but with distinct mechanisms of action. The essence of this approach lies in the detailed analysis provided by comparing the electrochemical signal responses of each probe to changes in analyte concentration. Such comparative analysis can be achieved by strategically adjusting the proximity of each probe to the electrode or by modulating their electrochemical responses through the introduction or removal of probes from the electrode surface in response to the presence of the target analyte. The ratio of signals from these two probes offers a method that is both highly sensitive and selective, making it particularly suitable for identifying and quantifying specific analytes in complex mixtures. In constructing these sensors, biomolecular techniques such as nucleic acid hybridization, aptamer recognition, and enzymatic cleavage play a crucial role. By harnessing these biomolecular interactions, dual-signal ratiometric electrochemical sensors can accurately detect and quantify target molecules with high precision.

## 3 Applications of ratiometric electrochemical sensors in food analysis

Ratiometric electrochemical sensors exhibit outstanding analytical performance and have promising prospects in food analysis. Based on the detection targets of ratiometric electrochemical sensors, they can be mainly divided into categories for detecting nutrients, additives, metal ions, pharmaceutical and pesticide residues, biotoxins, and pathogens. The following discussion will categorize and elaborate on these applications, underscoring the unique advantages and potential uses of ratiometric electrochemical sensors in ensuring food safety and quality.

### 3.1 Nutrients

Nutrients in food refer to essential and beneficial components that are crucial for assessing the quality of food products and medicinal ingredients (Shao, Hou, Jia, & Zheng, 2022; Similä, Ovaskainen, Virtanen, & Valsta, 2006). Accurate measurement of specific nutrient content is vital for ensuring the quality and safety of food items, as well as for understanding their nutritional value and health benefits. Ratiometric electrochemical sensors have proven particularly useful in the detection of specific types of nutrients such as quercetin and caffeic acid.

Quercetin (Qu) is emphasized as an important flavonoid due to its widespread occurrence in various plants and its significance in human diets (Kandemir, Tomas, McClements, & Capanoglu, 2022; Lai & Wong, 2022; Oh, Ambigaipalan, & Shahidi, 2021). Gui and Wang et al. have reported a direct ratiometric electrochemical method for detecting quercetin (Yu, Jin, Gui, Lv, & Wang, 2017). This assay employed thionine (TH) as the reference electroactive substance to analyze Qu. TH and Qu displayed distinct oxidation peaks at different potentials (−0.22 V for TH and 0.18 V for Qu), suitable for ratiometric measurements. Detection is based on differential pulse voltammetry (DPV), which measures the oxidation peak intensities of quercetin and thionine at specific voltages. The ratiometric method calculates the ratio of these peak intensities (*I*_Qu_/*I*_TH_), which linearly correlates with the concentration of quercetin in the solution. The sensor demonstrates high sensitivity and selectivity in detecting Qu, effectively overcoming potential interferences from real food samples.

Polyphenolic compounds are well-known for their excellent antioxidant properties, among which caffeic acid (CAE) is a key polyphenol, particularly abundant in chrysanthemum tea (Matsui, Tanaka, & Iwahashi, 2017; Naveed et al., 2018). Due to its stability, caffeic acid is often used as a benchmark for assessing the total content of polyphenolic compounds. Wang and Xie et al. have engineered a sophisticated ratiometric electrochemical sensor utilizing poly(methylene blue) (PMB) electropolymerized onto flower-like nickel-based metal-organic frameworks (Ni-TPA MOFs) for the precise detection of CAE (Yin, Zhuang, Xiao, Wang, & Xie, 2021). This sensor is built around a GCE that has been modified with a nanocomposite comprising PMB and Ni-TPA MOFs. Here, PMB acts as the internal reference probe, while Ni-TPA MOFs significantly boosts the electrochemical signal strength The sensor's functionality is based on a ratiometric electrochemical sensing strategy that measures the ratio of dual current peak intensities for CAE and PMB across different redox potentials. The sensor responds linearly across a CAE concentration range of 0.25–15.0 mM with a detection limit of 0.2 mM, and its accuracy has been corroborated against the traditional Folin–Ciocalteu method.

### 3.2 Additives

**Fig. 2:**
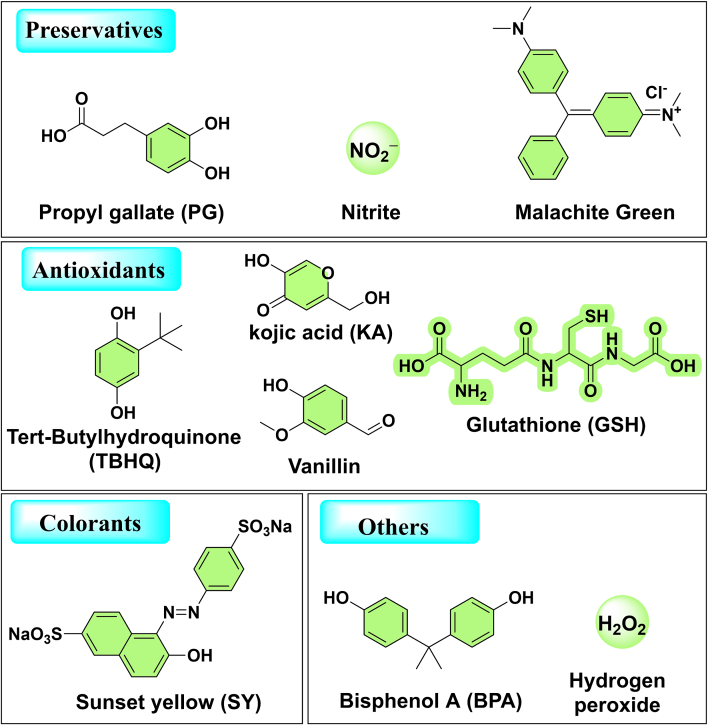
Schematic illustrations of the molecular structures of various types of food additives detected by currently reported ratiometric electrochemical sensors.

Food additives are substances added intentionally to food during various stages like processing, manufacturing, or storage to enhance appearance, texture, or preservation. Detecting food additives is crucial for ensuring food safety, compliance with regulatory standards, protecting consumer rights, enhancing public trust, and fostering the development of the food industry. Currently, ratiometric electrochemical sensors are widely used for the detection of various food additives (Fig. 2). These include preservatives such as propyl gallate, nitrites, and malachite green; antioxidants like vanillin, hydroquinone, kojic acid (KA), and glutathione; color additives; as well as substances that may remain in food from processing, such as bisphenol A and hydrogen peroxide (H_2_O_2_).

#### 3.2.1 Preservatives

Wang et al. developed a dual-ratiometric electrochemical sensor for detecting the food preservative propyl gallate (PG) (Yin, Wang, & Zhuang, 2021). The construction of the sensor involves anchoring anthraquinone (AQ) and multi-walled carbon nanotube (MWCNT) onto the GCE surface to leverage their excellent electrochemical activity and conductivity, enhancing the sensitivity for PG detection. As internal reference electroactive probes, AQ and MWCNT facilitate the display of three distinct current peaks corresponding to the electro-oxidation of PG, AQ, and MWCNT upon PG detection. The sensor quantifies PG through two ratiometric signals (*I*_PG_/*I*_AQ_ and *I*_PG_/*I*_MWCNT_), which linearly correlate with PG concentration ranging from 1.0 to 30.0 μM, with detection limits estimated at 0.67 μM and 0.76 μM, respectively. The sensor demonstrates satisfactory results in detecting PG in edible oil samples, showing high sensitivity and selectivity.

Nitrites are commonly used as preservatives and color enhancers in food processing. Due to the potential carcinogenic risk of nitrites reacting with amines to form nitrosamines and their ability to interfere with the transport of oxygen in the blood, monitoring nitrite levels in food is critical for ensuring food safety and public health. Zhou et al. developed a self-calibrating ratiometric electrochemical sensor for nitrite detection using uniformly dispersed silver nanoclusters (AgNCs) embedded in nitrogen-doped carbon sheets (NCS) (Yin et al., 2022). These AgNCs were evenly distributed within the NCS by in situ entrapment polymerization, utilizing an N-vinyl-functionalized imidazolium ionic liquid as a stabilizer and carbon precursor, thus optimizing the electrocatalytic performance. The oxidation peaks of nitrite and silver, the latter serving as a stable internal reference, were used as output signals. This sensor exhibited excellent sensing characteristics for nitrites, with a broad linear range (1.12–1400 μM), a low detection limit (0.38 μM), high stability, and strong anti-interference capabilities. Heng et al. introduced a ratiometric electrochemical sensor for detecting nitrites, utilizing the diazotization reaction between ortho-phenylenediamine (OPD) and nitrites (Niu, Wang, Hu, & Liu, 2022). Niu et al. reported a dual-mode ratiometric sensor for detecting nitrites, coupling diazotization with oxidase-mimetic catalysis using Mn_3_O_4_/TMB on carbon material, allowing colorimetric and electrochemical responses for improved accuracy in environmental and food matrices (Wang et al., 2022). Kan et al. developed a dual-mode colorimetric-electrochemical sensor for detecting nitrites, based on the specific diazotization reaction between nitrite and 3,3′,5,5′-tetramethylbenzidine (TMB) (Pan, Jiang, & Kan, 2023).

Malachite Green (MG), a dye and medicinal agent used in aquaculture, possesses antibacterial, antifungal, and parasiticidal properties but is a toxic triphenylmethane molecule associated with carcinogenic and teratogenic risks in humans. Zhou and Wang et al. developed a ratiometric electrochemical sensor using three-dimensional conductive metal-organic frameworks (CMOFs) to detect MG in fish samples (Li et al., 2023). The CMOFs, Cu_3_(HHTP)_2_, was synthesized through a one-pot process combining the ligand 2,3,6,7,10,11-hexahydroxytriphenylene (HHTP) with copper ions, with *N*,*N*-dimethylformamide (DMF) added as a limiting agent to control nanorod growth and morphology. Cu_3_(HHTP)_2_ functioned as an internal reference and displayed exceptional electrocatalytic activity and charge transfer efficiency, significantly enhancing the sensor's sensitivity to MG. Demonstrations showed that this electrochemical sensor offered a broad detection range (5–1000 nM) and a low detection limit of 1.34 nM, exhibiting excellent selectivity and interference resistance. Additionally, the sensor's application in actual fish samples validated its potential for food safety assurance.

#### 3.2.2 Antioxidants

**Fig. 3:**
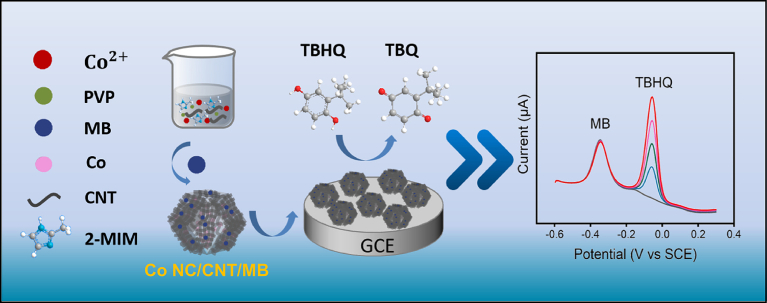
Schematic illustration of the construction and detection principle of a ratiometric electrochemical sensor for TBHQ based on Co NC/CNT/MB composite (Zhang, Liu, et al., 2024).

Tert-butylhydroquinone (TBHQ) is a synthetic antioxidant used primarily in edible oils to extend shelf life, but its excessive use can degrade food quality and pose health risks, leading to regulated maximum permissible levels. Wang et al. described the development of a ratiometric electrochemical sensor for detecting TBHQ (Cao, Wang, Zhuang, Wang, & Ni, 2019). The sensor utilizes a GCE modified with electrochemically deposited MnO_2_ and reduced graphene oxide (ERGO), forming the working electrode MnO_2_/ERGO/GCE. This method involves using two distinct signals at different potentials: one from TBHQ and the other from the internal MnO_2_ reference probe. The sensor exhibited two linear response ranges: 1.0–50.0 μM and 100.0–300.0 μM, with a detection limit of 0.8 μM. Ma et al. developed a ratiometric electrochemical sensor based on the self-assembly of Co NC/CNT and methylene blue for effective detection of TBHQ (Fig. 3) (Zhang et al., 2024). The sensor uses carbon nanotube-encapsulated Co/nitrogen-doped carbon (Co NC/CNT) and methylene blue (MB) as internal reference signals to enhance accuracy. The construction process includes self-assembling ZIF-67/CNT materials, which are then pyrolyzed in an argon atmosphere to form Co NC/CNT. This is followed by electrostatic adsorption to combine with MB, forming a Co NC/CNT/MB composite. Under optimized conditions, the ratio of the net peak currents of TBHQ to MB shows a linear relationship with TBHQ concentration. The sensor demonstrates excellent selectivity, repeatability, reproducibility, and stability, making it suitable for detecting TBHQ in real edible oil samples.

Vanillin is a widely used additive in food, cosmetics, and pharmaceuticals, primarily for its distinctive sweet aroma and antioxidant properties (Moradi, 2022). Jin and Gui et al. developed a novel ratiometric electrochemical aptasensor for the detection of vanillin that enhances sensitivity and selectivity through the use of advanced composite materials and biomolecular recognition elements (Sun, Jiang, Jin, & Gui, 2019). The sensor integrates Ketjen black and ferrocene dual-doped Metal-Organic Frameworks (ZIF-8, zeolite imidazole frameworks), combined with gold nanoparticles (AuNPs). A GCE is modified with these materials and a DNA aptamer that specifically binds to vanillin, creating a robust sensing platform. The method utilizes dual signal outputs from the target molecule vanillin and an internal reference (ferrocene incorporated into ZIF-8). The sensor operates within a vanillin concentration range of 10 nM to 0.2 mM, with a detection limit of 3 nM. The sensor has been successfully tested vanillin in real food samples (chocolate and nougat) e. Recently, Lee et al. developed a new sensor to detect the antioxidant kojic acid, structurally similar to vanillin, using an electrode modified with MXene/PB/AuNPs nanocomposites (Karuppaiah, Koyappayil, Go, & Lee, 2023).

Glutathione (GSH), a widely utilized food additive, is renowned for its potent antioxidant and detoxification properties (Tsiasioti, Zotou, & Tzanavaras, 2021). Liu et al. developed a highly sensitive and selective dual-signal, intrinsic self-calibrating electrochemical sensor for detecting GSH, leveraging the enhanced electrochemical properties of in situ electrodeposited silver nanoparticles on a nickel‑iron Prussian blue analog (Ag/NiFe PBA) (Xu et al., 2022). Two distinct oxidation peaks attributed to Ag and Fe species appear on the Ag/NiFe PBA-modified electrode. In the presence of chloride ions, Ag is oxidized to form silver chloride (AgCl), and subsequent addition of GSH triggers a specific interaction that significantly reduces the peak current of AgCl, while the peak current of NiFe PBA remains unchanged. The ratio of these peak currents (*I*_AgCl_/*I*_Fe_) serves as the signal output. The sensor exhibits an extraordinarily low detection limit of 0.12 nM. Successfully applied to various vegetable and serum samples, this sensor demonstrates its effectiveness and potential for broad applications in food safety and clinical diagnostics.

#### 3.2.3 Colorants

Food colorants, also referred to as food additives, are primarily utilized to enhance food colors, attracting consumers and improving product appearances. Sunset Yellow (SY), a synthetic azo dye commonly used in beverages, candies, desserts, and processed foods, has raised health concerns due to its potential allergenic and carcinogenic risks (Barciela, Perez-Vazquez, & Prieto, 2023). Sun and Xu et al. developed a ratiometric electrochemical sensor for SY using polydopamine (PDA) and nickel sulfide on hollow carbon spheres (NiS@HCS) as primary materials (Chen et al., 2022). The NiS@HCS was synthesized via a hydrothermal method to enhance its catalytic activity and conductivity. Polydopamine served as an internal reference signal, forming a film on the GCE through electropolymerization, enhancing the sensor's sensitivity and selectivity toward SY. The sensor quantifies SY based on the ratio of electrochemical signals from SY and PDA, where PDA serves as an internal reference. The sensor was tested in real food samples such as rice vinegar, cooking wine, and carbonated drinks, showing good recovery rates and consistency with UV–Vis spectroscopy. Subsequently, Wu, Zhang, and Ma et al. developed a gold nanoparticle-based ratiometric molecularly imprinted electrochemical sensor for SY (Wu et al., 2023). Compared to traditional sensors, molecularly imprinted biosensors offer higher specificity and sensitivity due to the specific recognition of template molecules by molecularly imprinted polymers (MIPs). This sensor is not affected by other structurally similar interferents and performs well in the analysis of real samples.

#### 3.2.4 Others

Other substances primarily include residuals from food processing or storage, such as bisphenol A (BPA) and hydrogen peroxide (H_2_O_2_). Bisphenol A (BPA), used in polycarbonate plastics and epoxy resins, is an environmental endocrine disruptor that mimics estrogen and can cause reproductive, developmental, and other health issues (Beitollahi et al., 2022). Wang et al. developed a ratiometric electrochemical sensor for detecting BPA (Sun, Xiao, Tang, Zhuang, & Wang, 2021). This sensor uses a GCE modified with a composite of polythionine (PTH) and gold nanoparticles (AuNPs). The ratiometric strategy leverages the ratio of oxidation peak currents between BPA and the internal reference PTH for self-calibration, enhancing the reliability of the detection. The sensor also exhibited high sensitivity, stability, and selectivity, demonstrating its potential for practical application in real water samples. Pang et al. described a ratiometric electrochemical sensor for simultaneously detecting BPA and bisphenol S (BPS), using a carbon cloth electrode modified with a covalent organic framework (COF-LZU1) and AgNPs (Pang, Wang, Shen, & Qiao, 2022).

Hydrogen peroxide (H_2_O_2_) is used in the food industry as a disinfectant and bleaching agent to control microorganisms and maintain food color and texture, but its residues must be monitored due to potential health hazards like irritation and oxidative stress that can cause cellular damage (Abdelshafy, Hu, Luo, Ban, & Li, 2024; Xing et al., 2022). Kan et al. developed a ratiometric electrochemical sensor for the quantitative detection of H_2_O_2_ in food samples (Luo & Kan, 2022). The sensor is based on the specific dissociation reaction of 4-aminophenylboronic acid ester (ABAPE) triggered by H_2_O_2_ to generate electroactive 4-aminophenol (4-AP), providing high selectivity. The sensor uses polythiophene (TH) as the reference probe and is modified with Ketjen black (KB) and gold nanoparticles (AuNPs) on the electrode surface, enhancing the electrocatalytic activity toward 4-AP. The sensor measures the ratio of 4-AP to TH currents, which correlates well with H_2_O_2_ concentrations. Moreover, the ratiometric strategy sensor demonstrates good accuracy, reliability, and stability, successfully detecting H_2_O_2_ in milk, beer, and juice samples with satisfactory results. Following that, Li et al. described a ratiometric electrochemical sensor based on a ZIF-8/COOH-MWCNTs composite specifically designed for the detection of H_2_O_2_ (Zhao et al., 2023). The oxidation peak current of MB serves as the internal reference signal, while the response electrochemical signal is generated from the oxidation peaks of 4-AP, produced by the reaction of H_2_O_2_ with ABAPE.

### 3.3 Metal ions

Monitoring heavy metals is an essential task in food testing, and ratiometric electrochemical sensors have also been used for monitoring Cd^2+^ and Pb^2+^. Zhang et al. developed a dual-signal ratiometric electrochemical sensor based on semi-complementary aptamers for detecting cadmium in mussels (Chen, Liu, Su, Zhang, & Zou, 2021). This sensor utilizes a homemade multi-functional screen-printed electrode (SPE) that enables both primary ratiometric sensing and auxiliary on-chip pH monitoring. The preparation process of the sensor involves electrodepositing AuNPs onto the SPE. The Cd^2+^-specific aptamers are then attached to the AuNPs through Au—S bonds to form Apt/AuNPs/SPE. Next, ssDNA@PTH-Au (reference probe) is dropped onto the electrode, where it hybridizes with the Cd^2+^ aptamer to produce ssDNA@PTh-Au/Apt/AuNPs/SPE. The binding of Cd^2+^ to its specific aptamer changes the aptamer's configuration and expels the previously hybridized ssDNA@PTH-Au, resulting in a reduction in the reference signal. Meanwhile, the signal for Cd^2+^ increases. This change forms a “signal on/off” type of ratiometric detection system where the target signal (*I*_Cd_) and the reference signal (*I*_PTH-Au_) constitute a ratiometric sensing system. The sensor demonstrates a detection range from 2 × 10^−3^ to 8 × 10^−1^ mg∙L^−1^, with a detection limit of 7 × 10^−1^ mg∙L^−1^. This sensor has been successfully applied to the determination of cadmium in actual mussel samples, showing satisfactory recovery results, confirming its practicality and accuracy.

Zhang et al. developed a sensor employing a composite electrode composed of ZrO_2_, Ni/Co-MOFs, and AuNPs, which was modified with hairpin-structured nucleic acid chains tagged with anthraquinone-2-carboxylic acid (AQ) (Wang et al., 2023). The sensor uses two target-specific aptamer strands, each marked with an electrochemical probe ferrocene (Fc) for Cd^2+^ and MB for *S. aureus*, enabling simultaneous detection. This ratiometric sensor operates by measuring the ratio of the MB and AQ signals to the Fc signal, effectively leveraging the redox potential differences between these markers to achieve precise and specific detection of both targets. Zou et al. developed a ratiometric electrochemical sensor based on the synergistic action of semi-complementary aptamer pairs and Ag nanowires@zeolitic imidazolate framework-8 (AgNWs@ZIF-8) for detecting Pb^2+^ in fish (Han et al., 2023). Dai et al. developed a sensor composed of curcumin (p-CCM), MWCNTs, and layered double hydroxides (LDHs), forming a novel ratiometric electrochemical sensing interface for Cd^2+^ and Pb^2+^ (Duan et al., 2024).

### 3.4 Pharmaceutical and pesticide residues

**Fig. 4:**
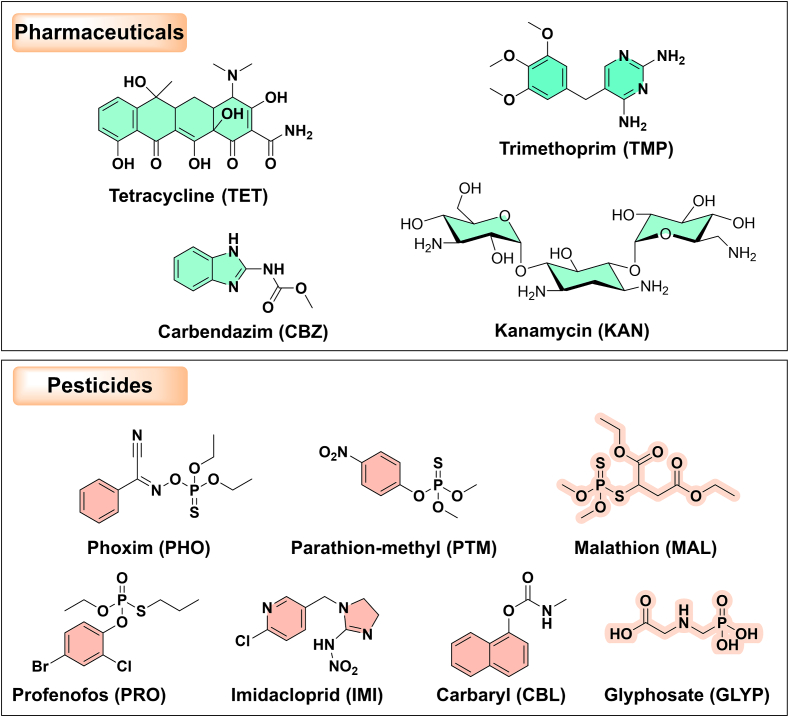
Schematic illustrations of the molecular structures of various pharmaceuticals and pesticides detected by currently reported ratiometric electrochemical sensors in food products.

Pharmaceutical and pesticide residues are chemicals that can remain in food products due to agricultural activities and pharmaceutical applications. These substances can have profound health implications, including fostering antibiotic resistance, disrupting hormonal functions, and causing other toxicological impacts. Therefore, it is essential to detect and quantify these residues to ensure consumer safety and meet regulatory standards. Ratiometric electrochemical sensors have proven to be highly effective for this purpose. Such sensors have already been applied to detect a range of pharmaceutical residues (like tetracycline, kanamycin, trimethoprim, and carbendazim) and pesticide residues (such as imidacloprid, carbaryl, parathion-methyl, phoxim, malathion, profenofos, and glyphosate) (Fig. 4).

#### 3.4.1 Pharmaceutical residues

Tetracycline (TET) is a broad-spectrum antibiotic widely used in animal husbandry, which can leave residues in animal-derived food products, potentially causing health issues such as allergic reactions and antimicrobial resistance (Khezerlou et al., 2023; Wang, Yin, & Gunasekaran, 2023). Guo and Zhang et al. developed a separable ratiometric electrochemical sensor designed effectively for detecting TET residues (Xu et al., 2017). This innovative sensor comprises two distinct aptasensors: Aptasensor 1 utilizes screen-printed carbon electrodes (SPCEs) that are electrodeposited with AuNPs to create a foundational layer for the attachment of TET-specific aptamers via Au—S bonds; Aptasensor 2 similarly incorporates carbon nanofibers (CNFs) combined with AuNPs on SPCEs, where TET-specific aptamers are also attached. Upon exposure to TET, the aptamers bound to TET undergo a spatial conformation change, affecting the electrochemical characteristics of the nanocomposites. This alteration leads to measurable changes in the electrical current at each aptasensor. The ratiometric measurement (Δ*I*_CNFs_/Δ*I*_Fc_, where Δ*I*_CNFs_ and Δ*I*_Fc_ represent the current changes in Aptasensor 2 and Aptasensor 1, respectively) ensures precision. The sensor exhibits a detection range for TET from 10^−8^–10^−3^ g∙L^−1^, with a detection limit of 3.3 × 10^−7^ g∙L^−1^, and has been successfully validated in real milk samples. Kanamycin (KAN) is an aminoglycoside antibiotic widely used in animal husbandry, and its residues can pose health risks such as antibiotic resistance (Tang & Tao, 2023). Li and Xu et al. developed a ratiometric electrochemical aptasensor for detecting KAN using a Fc-labeled primer and a graphdiyne-methylene blue (GDY-MB) nanocomposite (Gao et al., 2022). The sensor operates by forming a hairpin structure when KAN binds to the aptamer, enhancing the ferrocene signal near the electrode surface. Exonuclease I is used to increase the signal through cyclic amplification. This aptasensor demonstrates high stability and specificity, effectively detecting KAN in milk samples. Subsequently, Jin and colleagues developed an enzyme-free ratiometric electrochemical aptasensor for detecting KAN in food (Jin et al., 2024). Utilizing an entropy-driven strand displacement reaction, the sensor demonstrated high specificity, reproducibility, and remarkable sensitivity. Deng et al. developed a molecularly imprinted ratiometric electrochemical sensor for sensitive and selective detection of trimethoprim (TMP), another broad-spectrum antibiotic (Deng et al., 2024). The sensor utilizes a 3D—1D hetero-nanoflower structure composed of MoS_2_ and carbon nanotubes as the substrate, which enhances electrical conductivity and provides a larger surface area to optimize sensor performance. The sensor employs a molecularly imprinted polymer for specific recognition and quantification of TMP, using ferrocene as a reference for ratiometric measurement.

Carbendazim (CBZ) is a broad-spectrum fungicide extensively used in agriculture, known to cause potential chromosomal abnormalities and environmental hazards (Wang, Shi, Liu, & Laborda, 2020; Zhou, Guo, Wang, Wang, & Zhang, 2023). Gao and Lu et al. developed a ratiometric electrochemical sensor for detecting CBZ, which is constructed from a composite of MXene@Ag nanoclusters and amino-functionalized multi-walled carbon nanotubes (NH_2_-MWCNTs) (Zhong et al., 2021). This combination enhances the sensor's electrocatalytic capabilities, and the presence of Ag nanoclusters provides a stable reference signal essential for the ratiometric detection method. The addition of NH_2_-MWCNTs improves the electrochemical signals of CBZ and Ag, leading to signal amplification and increased sensitivity. The sensor demonstrates a good linear relationship between the ratio of CBZ to Ag signal intensities (*I*_CBZ_/*I*_AgNCs_) and the concentration of CBZ, ranging from 0.3 nM to 10 μM, with a detection limit of 0.1 nM.

#### 3.4.2 Pesticide residues

Current advancements in ratiometric electrochemical sensors are predominantly focused on detecting organophosphorus pesticides (OPs), such as phoxim, parathion-methyl, malathion, and profenofos. These pesticides are known for their broad-spectrum insecticidal effects and relatively rapid action, primarily inhibiting acetylcholinesterase (AChE), which can be potentially toxic to both the environment and non-target organisms, including humans (Sarlak et al., 2021; Wan, Liu, Pi, & Wang, 2023).

**Fig. 5:**
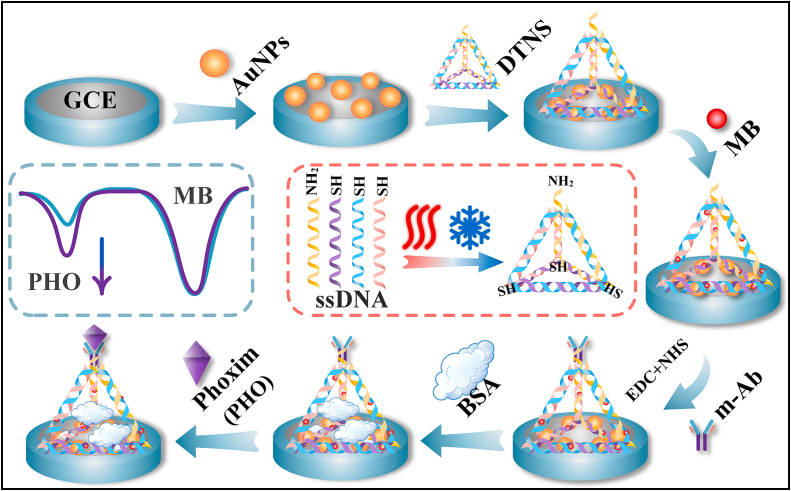
Schematic illustration of the construction and detection principle of a ratiometric electrochemical immunosensor for phoxim (Su, Wang, et al., 2022).

Zhang et al. have developed a ratiometric electrochemical immunosensor utilizing DNA tetrahedral nanostructures (DTNS) for detecting phoxim (PHO) in vegetables (Fig. 5) (Su et al., 2022). The sensor is constructed by modifying a GCE with AuNPs and anchoring DTNS via gold‑sulfur (Au—S) bonds. MB, absorbed onto the DTNS, forms the MB/DTNS/AuNPs/GCE substrate. The DTNS adheres spontaneously to the modified electrode, creating a stable three-dimensional structure that offers numerous binding sites for the internal reference signal probe, MB. A monoclonal antibody (m-Ab) is vertically linked to the apex of the DTNS, selectively responding to the antigenic phoxim and enhancing the target signal. This configuration establishes a ratiometric index, *I*_Phoxim_/*I*_MB_, through the DPV technique. The sensor exhibits a robust linear response in the range of 0.1–30 μg∙L^−1^ and a detection limit of 3 ng∙L^−1^. Then, the Zhang group developed a novel ratiometric electrochemical immunosensor for the detection of parathion-methyl (PTM) (Su et al., 2022). Initially, AuNPs were electrodeposited onto a SPE, which was then modified with antibodies targeting PTM. MXene-Au nanocomposites were synthesized via a hydrothermal method, and MB, serving as the reference signal probe, was adsorbed onto MXene-Au through electrostatic adsorption. Subsequently, MXene-Au-MB was conjugated with the PTM antigen (ATG) to form a competitive signal probe complex, MXene-Au-MB-ATG. During detection, PTM competes with MXene-Au-MB-ATG for binding to the SPE. The presence of PTM causes partial dissociation of MXene-Au-MB-ATG from the antibodies, altering the sensor's current signal. The sensor utilizes a dual-electric-field mode, enhanced by DC-biased sinusoidal excitations, to amplify the immunoreaction.

Guo and Liu et al. introduced a dual-ratiometric electrochemical aptasensor for the simultaneous detection of two organophosphorus pesticides, malathion (MAL) and profenofos (PRO) (Li et al., 2023). This sensor integrates an innovative hairpin tetrahedral DNA nanostructure (HP-TDN) with metal ions and nanocomposites, building a complex sensory framework that enhances signal amplification. The sensor's architecture utilizes AuNPs embedded in a zeolitic imidazolate framework (ZIF-8) to form an Au@ZIF-8 nanocomposite, significantly boosting conductivity and biocompatibility. The HP-TDN structure, tagged with TH, provides specific binding sites for the Pb^2+^ and Cd^2+^ tags linked to the MAL and PRO aptamers. When MAL and PRO are present, their corresponding aptamers bind to these analytes and dissociate from HP-TDN's complementary strands, leading to reduced oxidation currents for Pb and Cd. Thionine's oxidation current remains stable and serves as a reliable reference, enabling ratiometric measurements. The ratios of the oxidation currents (*I*_Pb_/*I*_TH_ and *I*_Cd_/*I*_TH_) are used to quantify the concentrations of MAL and PRO.

Ratiometric electrochemical sensors are also employed to detect various other types of pesticides, including the neonicotinoid imidacloprid (Liu et al., 2021; Zhang et al., 2020), the carbamate carbaryl (Zhang, Zhang, et al., 2020), and the herbicide glyphosate (Zhao, Guo, Lan, & Liu, 2024). Zou et al. developed a novel signal on-off ratiometric electrochemical sensor to detect imidacloprid (IMI), enhancing selectivity and stability through molecularly imprinted polymer (MIP) technology (Zhang et al., 2020). The sensor uses 6-(Ferrocenyl)hexanethiol (FcHT) as an internal reference signal and creates an MIP film on a GCE through electropolymerization. This film specifically recognizes IMI by its shape and chemical functionalities. Initially, AuNPs are electrodeposited on the GCE to expand the area available for FcHT and MIP attachment. Subsequently, FcHT is self-assembled in a solution to form an FcHT/AuNPs layer. An MIP layer is then formed over this layer in the presence of IMI template molecules by electropolymerizing *o*-phenylenediamine, followed by acid treatment to remove the template molecules, thus creating a functional MIP/FcHT/AuNPs/GCE electrode. Under optimal conditions, the sensor exhibits a linear response range from 0.5 to 100 μM and a detection limit of 47 nM. Later, the same research group developed an IMI ratiometric electrochemical sensor based on a flexibly fabricated vibratory electrode module (Liu et al., 2021). This construction utilizes a composite modification of AuNPs, Prussian Blue (PB), and β-cyclodextrin (β-CD), with PB serving as the internal reference signal and β-CD as the molecular recognizer.

### 3.5 Biotoxins

**Fig. 6:**
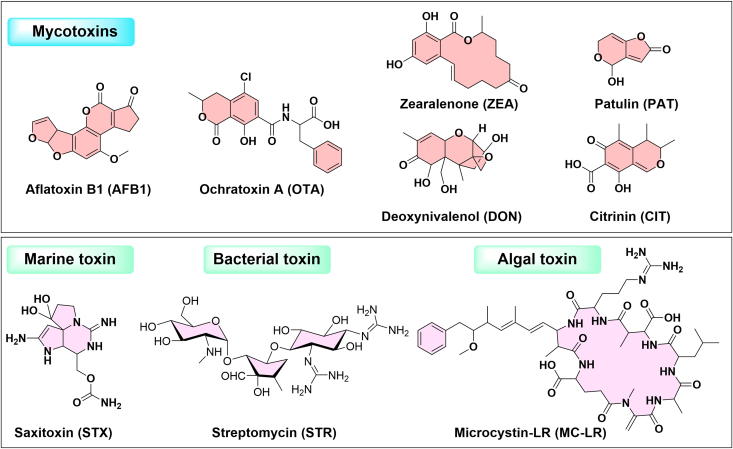
Molecular structures of various biotoxins detected by currently reported ratiometric electrochemical sensors in food products.

Biotoxins are harmful substances produced by microorganisms, plants, or animals that can accumulate in food and water, posing significant health risks to humans. Monitoring these toxins is essential for food safety and public health because they can cause a variety of health issues including acute and chronic poisoning, neurological damage, immune suppression, and cancer. Ratiometric electrochemical sensors have been effectively employed to detect various biotoxins (Fig. 6). These include mycotoxins such as aflatoxins, ochratoxin A, zearalenone, patulin, deoxynivalenol, and citrinin; marine toxins like saxitoxin; bacterial toxins such as streptomycin; algal toxins including microcystin; and plant toxins like the Cry1Ab protein.

#### 3.5.1 Mycotoxins

Aflatoxin B1 (AFB1), produced by molds, is a highly toxic chemical carcinogen that can contaminate a variety of food products including grains and peanuts (Chen, Abdallah, Chen, & Rajkovic, 2023; Diao, Hou, & Dong, 2013; Jia et al., 2022; Liu et al., 2022). Han et al. developed a ratiometric electrochemical and electrochemiluminescence (ECL) aptasensor for detecting AFB1 in food (Wu et al., 2017). In the electrochemical system, the sensor design utilizes a Fc-labeled AFB1 aptamer probe and MB-labeled complementary DNA, providing dual signals. Upon specific binding with its aptamer, the conformational change in the aptamer causes the MB-labeled DNA strand to detach from the electrode surface, enhancing the signal from the Fc label while diminishing the MB signal. This “signal-on/off” mode simplifies the sensor's signal reading process and enhances the accuracy and sensitivity of detection. In the ECL system, CdTe/CdS/ZnS quantum dots and luminol serve as the signal sources, with horseradish peroxidase-modified gold nanorods acting as the quencher/enhancer. In the absence of AFB1, cDNA hybridizes with the aptamer, maintaining both ECL signals. When AFB1 is present, the aptamer binds with the target and releases the cDNA sequence, leading to an increase in the ECL signal from the quantum dots and a decrease from the luminol.

**Fig. 7:**
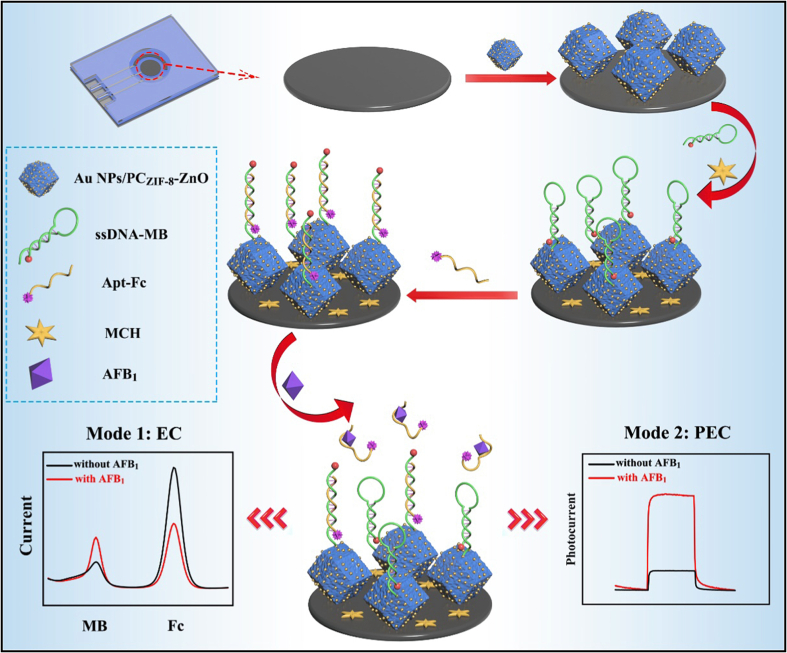
Schematic illustration of the construction and detection principle of a ratiometric electrochemical and photoelectrochemical dual-mode aptasensor for AFB1 (Zhang, Li, et al., 2024).

Using dual-detection modalities to construct a ratiometric sensing system is a highly effective strategy. Fang et al. developed an innovative ratiometric sensing approach based on ECL and electrochemical methods for detecting AFB1 (Lv et al., 2022). This strategy incorporates a newly synthesized ECL emitter, BPYHBF, integrated with a composite material of multi-walled carbon nanotubes (MWCNTs) within a ferrocene-modified phenolic resin metal-organic framework (Fc-MOF). The sensor employs a competitive immunoassay format, where BPYHBF serves as a luminescent tag competing with the target antigen. This configuration provides robust ECL signals in the presence of tri-n-propylamine. Additionally, the MWCNTs/Fc-MOF is used as the sensing base to enhance the internal reference electrochemical signal, thus allowing for self-calibration to enhance sensitivity and reliability. Recently, Feng and Cao et al. developed a dual-mode electrochemical/photoelectrochemical (EC/PEC) aptasensor for the highly sensitive detection of AFB1 (Fig. 7) (Zhang et al., 2024). The sensor was designed using a novel composite material, Au NPs/PC_ZIF-8_-ZnO, synthesized from ZIF-8 precursors integrated with ultrasmall gold nanoparticles (Au NPs) to enhance the sensing substrate. Thiolated MB-labeled single-stranded DNA (ssDNA-MB) was anchored to the composite via Au—S bonds. This ssDNA-MB hybridized with a Fc-labeled aptamer (Apt-Fc) to form double-stranded DNA (dsDNA). In the presence of target AFB1, the dsDNA dissociates due to the specific binding of AFB1 to Apt-Fc, leading to the detachment of the Fc label from the electrode surface and the reformation of the hairpin structure of ssDNA-MB. This causes a decrease in the Fc oxidation peak current and an increase in the MB oxidation peak current in the EC mode, facilitating ratiometric electrochemical detection. Additionally, the sensitization effect of MB enhances the photoelectrochemical current response of the electrode, allowing for a “signal off-on” PEC detection mode. Liu and You et al. utilized a hybridization chain reaction (HCR) to amplify the detection signal, using MB and Fc as electroactive indicators to generate response and reference signals, respectively, constructing a label-free, homogeneous electrochemical aptasensor for detecting AFB1 in grains (Zhu, Liu, Li, Chen, & You, 2022). Additionally, other nucleic acid technologies such as exonuclease I (Exo I) and a DNAzyme-driven tripedal DNA walker were also employed to build ultra-sensitive ratiometric electrochemical sensors for AFB1 (Cui et al., 2022; Liang et al., 2024; Lv et al., 2023; Wang, Qian, An, Lu, & Huang, 2019).

Ochratoxin A (OTA), produced by fungi like Aspergillus and Penicillium and found in grains and various foods, is monitored for its kidney damage, immunosuppression, and potential carcinogenic effects to ensure public health safety (Schrenk et al., 2020). In 2018, Wang and Lin et al. developed a highly reproducible ratiometric aptasensor that combines ECL and electrochemical signals to detect OTA (Lin et al., 2018). This sensor design utilized two probes: a Ru(phen)_3_^2+^ labeled working probe (Ru(phen)_3_^2+^-wp) generating the ECL signal, and a MB labeled reference probe (MB-rp) generating the electrochemical signal. Liu and You et al. reported a ratiometric electrochemical aptasensor for detecting OTA, employing dual signal amplification through MB and Fc-tagged DNA. The specific binding of OTA induces changes in DNA structure, altering the electrochemical signals on the sensor, enabling highly sensitive and precise detection of OTA (Zhu et al., 2020). Utilizing DNA strands labeled with MB and Fc, as well as nucleic acid hybridization and strand displacement reactions, other highly sensitive ratiometric electrochemical sensors have also been developed for the detection of OTA (Liang et al., 2021; Liu et al., 2023).

Recently, novel nanostructured materials have been successfully employed to develop electrochemical sensors for detecting OTA. Yang and Hu et al. developed a sensor based on DNA tetrahedral nanomaterial (NTH), combined with zirconium metal-organic framework (UiO-66) as a signal tag for the detection of OTA (Li et al., 2022). In this sensor, UiO-66 and an electrolyte solution of [Fe(CN)_6_]^3−/4−^ are utilized as the signal probe and the internal reference probe, respectively. Wu and Hu et al. reported a novel dual-signal ratiometric electrochemical aptasensor based on cobalt metal-organic frameworks (Co-MOFs) for the detection of OTA in foods (Guan et al., 2023). This biosensor utilizes Co-MOFs as the signal probe and MB as the internal reference probe, demonstrating high sensitivity and reproducibility. The sensor design incorporates DNA walker technology along with a cyclic nucleic acid enzymatic cleavage process to amplify the detection signal for OTA. Recently, Wang et al. developed a novel high-sensitivity dual-signal ratiometric electrochemical aptasensor for OTA, utilizing nanoporous gold (NPG) (Wang et al., 2024). The high surface area and conductivity of NPG facilitate the attachment of a greater number of aptamers, significantly amplifying the signals. Furthermore, Suo and Wei et al. developed a ratiometric electrochemical aptasensor for detecting ZEN in corn flour, using MB and Ag^+^ as dual-signal probes, enhanced with Au@Pt/Fe-N-C nanocomposites (Suo et al., 2022). This design significantly improves the sensor's sensitivity due to the large surface area and high conductivity of the nanocomposites.

Antigen-antibody recognition and molecular imprinting technologies have also given rise to other ratiometric electrochemical sensors for detecting OTA. Xu and Liu et al. developed a versatile immunosensor capable of detecting OTA, AFB1, and zearalenone (ZEN) (Qileng et al., 2020). This sensor combines PEC and electrochemical signal responses, utilizing the PEC signals generated by cadmium sulfide nanoparticles under light-activated conditions and signals produced by CuS electrochemical reactions for dual validation and ratiometric detection of target analytes. Additionally, the same research group introduced a novel ratiometric immunosensor for distinguishing different ochratoxins in mixtures (Qileng et al., 2020). Using the molecular imprinting technology, Zeng et al. developed another Ratiometric sensor with enhanced specificity for detecting OTA (Hu, Xia, Liu, Chen, & Zeng, 2022).

Ratiometric electrochemical sensors have been developed to detect other mycotoxins such as patulin, deoxynivalenol, and citrinin. Wang and Liu et al. have detailed a dual-mode aptasensor combining EC and PEC methods for the ratiometric detection of patulin (PAT) (Liu et al., 2023). This sensor utilizes CdTe quantum dots sensitized with gold nanorods (CdTe QDs/Au NRs) to generate photocurrent, while a ferrocene (Fc)-tagged aptamer produces redox current. The quantitative detection of PAT is achieved by the ratio of PEC to EC signals. Dai et al. developed a ratiometric electrochemical immunosensor based on iron-based metal-organic frameworks (Fe-MOF) and gold nanoparticles (AuNP) for the detection of deoxynivalenol (DON) in cereal products (Shu et al., 2024). The sensor uses Fe-MOF/AuNP as the signal probe combined with immunoglobulin G (IgG)-tagged, and [Fe(CN)_6_]^3−/4−^ as the internal reference probe, forming a competitive capture and detection system for DON. Zeng et al. developed a novel ratiometric electrochemical sensor for selective detection of citrinin (Hu, Liu, Xia, Zhao, & Zeng, 2021). Poly(thionine) serves a dual role as both the MIP and the reference probe, while [Fe(CN)_6_]^3−/4−^ acts as the indicator probe, whose signal decreases upon addition of citrinin, allowing for ratiometric measurement of citrinin levels.

#### 3.5.2 Other biotoxins

Ratiometric electrochemical sensors have also been developed for detecting other biotoxins such as marine toxin (saxitoxin), bacterial toxin (streptomycin), algal toxin (microcystin), and plant toxin (Cry1Ab protein). Wu et al. developed a highly sensitive aptamer-based electrochemical sensor for detecting marine biotoxin saxitoxin (STX) (Zeng, Tang, Wu, Han, & Wu, 2024). This sensor utilizes silver nanoparticles (Ag NPs) regulated by K_3_Fe(CN)_6_ as a source of dual electrochemical signals. Xu et al. constructed a highly sensitive ratiometric electrochemical sensor for detecting streptomycin (STR), utilizing a dual amplification mechanism of functionalized gold nanoparticles (AuNPs) and hybridization chain reaction (HCR) (Zhang, Jia, & Xu, 2023). Li et al. developed a ratiometric electrochemical sensor for detecting microcystin-LR (MC-LR) in aquatic products, emphasizing a novel dual-signal strategy combining methylene blue-tagged single-stranded DNA (MB-ssDNA) and ferrocene-tagged single-stranded DNA (Fc-ssDNA) (Li, Li, Zhao, Wang, & Li, 2022). Liu and You et al. developed a novel in-situ ratiometric PEC immunosensor for detecting Cry1Ab protein (Meng et al., 2022). This sensor adopts a sandwich-type PEC structure, with gold nanorods sensitized quantum dots fixing the primary antibody (Au NRs/QDs-Ab1), and methylene blue sensitized quantum dots binding the secondary antibody (MB/QDs-Ab2), serving as the photoelectric base layer and signal amplifier, respectively.

### 3.6 Pathogens

Pathogens, including bacteria, viruses, fungi, and parasites, can contaminate food and water, leading to illnesses ranging from mild gastroenteritis to severe diseases like meningitis and sepsis. Due to their high sensitivity and specificity, ratiometric electrochemical sensors have been developed to detect pathogens, specifically targeting organisms like Salmonella and *Staphylococcus aureus*.

Salmonella is a common bacterium that can be transmitted through consuming contaminated food or water, causing symptoms such as diarrhea and fever, and requiring antibiotics in severe cases. Currently, there are several studies on ratiometric electrochemical sensors for detecting Salmonella, which specifically target biomarkers such as Salmonella esterase and characteristic nucleic acids. Huang et al. developed two ratiometric electrochemical probe molecules, named Sal-CAF and Sal-NBAF, based on ferrocene moieties specifically for detecting Salmonella esterase (Kumaravel, Jian, Huang, Huang, & Hong, 2022). The octyl esters in Sal-CAF can be cleaved by the Salmonella esterase, initiating a trimethyl lock mechanism that releases electron-rich aminoferrocene derivatives. This action shifts the DPV peak potential from 0.29 V to −0.08 V (vs Ag/AgCl), facilitating a ratiometric response. Furthermore, the same research group enhanced the sensitivity for detecting Salmonella esterase by combining the highly selective probe Sal-CAF with an electrode modified with graphene quantum dots‑gold nanoparticles, achieving a detection limit of 35.62 × 10 CFU∙mL^−1^ (Kumaragurubaran et al., 2023). Yang and Zhang et al. developed a novel ratiometric electrochemical biosensor designed for rapid and sensitive detection of *Salmonella enterica* serovar Typhimurium in food products (Yu, Yuan, Zhang, Guo, Lu, Yang, et al., 2022). This biosensor utilizes saltatory rolling circle amplification (SRCA) paired with a dual-signal electrochemical readout to improve both specificity and sensitivity. It features an electrode surface immobilized with mercapto-modified β-cyclodextrin and Au NPs, providing a robust platform for SRCA reactions. Subsequently, the same research group combined SRCA with the CRISPR/Cas12a system to develop a novel ratiometric electrochemical biosensor for the ultrasensitive and specific detection of Salmonella (Zheng et al., 2023). By effectively integrating rapid SRCA amplification and the trans-cleavage capabilities of Cas12a, signal amplification was achieved, thereby eliminating non-specific amplification. The sensor was able to detect Salmonella at concentrations as low as 2.08 fg∙μL^−1^ in pure cultures.

*Staphylococcus aureus* (*S. aureus*) is a common pathogen that can cause various infections, ranging from minor skin infections to life-threatening diseases such as pneumonia, meningitis, and sepsis. Wu and Yang et al. developed a dual-mode ratiometric aptasensor based on DNAzyme activation recycling for the ultrasensitive and accurate detection of *S. aureus* (Shan, Kuang, Feng, Wu, & Yang, 2023). This sensor utilized ECL and electrochemical signals for dual-mode detection to achieve a ratiometric response to the target. Specifically, the probe DNA labeled with an ECL emitter (probe 2-Ru) contains a blocked DNAzyme and is partially hybridized with the aptamer, which is then captured by the probe DNA labeled with an EC indicator (probe 1-MB) on the electrode surface. When *S. aureus* is present, the conformational change of probe 2-Ru activates the blocked DNAzymes, leading to the recycling cleavage of probe 1-MB, bringing the ECL tag closer to the electrode surface. Due to the reverse change tendencies of the ECL and EC signals, the sensor achieves quantitative detection of *S. aureus*. Meanwhile, the same research group developed another highly sensitive ratiometric electrochemical sensor for detecting *S. aureus* based on dual DNA recycling amplification and Au NPs@ZIF-MOF-modified electrodes (Shan, Xie, Zhou, Wu, & Yang, 2023).

## 4 Conclusions and perspectives

Ratiometric electrochemical sensors represent an advanced electroanalytical method that effectively overcomes the background noise issues inherent in traditional electrochemical sensors by utilizing the ratio of signals from multiple electrochemically active substances, thus enhancing measurement accuracy and repeatability. This review comprehensively discusses the application of ratiometric electrochemical sensors in food analysis, assessing their unique advantages in food safety and quality detection. We have explored the two main types of ratiometric electrochemical sensors: the internal reference type and the dual-signal response type. Moreover, the review highlights emerging methods for enhancing sensor specificity using antibody recognition, aptamer binding, and molecularly imprinted polymers, as well as signal amplification techniques involving nucleic acid technologies and nanomaterials, opening new pathways to improve sensor sensitivity and selectivity.

Despite the widespread attention ratiometric electrochemical sensors have received in food analysis in recent years and the significant progress made, several challenges remain, such as the complexity of sensor design and manufacturing, the lack of effective in situ real-time analysis, and the limited range of detectable targets. Future developments could focus on several directions: (1) Developing electrode base composite functional materials with more controllable structure and performance that not only provide stable reference signals but also catalyze target reactions to generate response signals, thereby simplifying sensor construction and operation; (2) Constructing ratiometric electrochemical sensors for food analysis based on microelectrodes, utilizing the characteristic of in situ analysis to facilitate real-time monitoring of food; (3) Developing detection systems based on responsive organic electrochemical probes, where organic small molecules with precise structures and adjustable properties, coupled with different recognition groups, could allow the use of single probe molecules to conveniently detect various analytes.

## CRediT authorship contribution statement

**Xincheng Hu:** Writing – original draft, Investigation, Funding acquisition, Conceptualization. **Wei Wei:** Writing – review & editing, Supervision, Conceptualization. **Xinyi Li:** Writing – review & editing. **Yewen Yang:** Writing – review & editing, Visualization. **Binbin Zhou:** Writing – review & editing, Supervision, Project administration, Funding acquisition.

## Declaration of competing interest

The authors declare that they have no known competing financial interests or personal relationships that could have appeared to influence the work reported in this paper.

## Data Availability

No data was used for the research described in the article.

## References

[bb0005] Abdelshafy A.M., Hu Q., Luo Z., Ban Z., Li L. (2024). Hydrogen peroxide from traditional sanitizer to promising disinfection agent in food industry. Food Reviews International.

[bb0010] Ayerdurai V., Cieplak M., Kutner W. (2023). Molecularly imprinted polymer-based electrochemical sensors for food contaminants determination. TrAC Trends in Analytical Chemistry.

[bb0015] Barciela P., Perez-Vazquez A., Prieto M.A. (2023). Azo dyes in the food industry: Features, classification, toxicity, alternatives, and regulation. Food and Chemical Toxicology.

[bb0020] Beitollahi H., Shahsavari M., Sheikhshoaie I., Tajik S., Jahani P.M., Mohammadi S.Z., Afshar A.A. (2022). Amplified electrochemical sensor employing screen-printed electrode modified with Ni-ZIF-67 nanocomposite for high sensitive analysis of Sudan I in present bisphenol A. Food and Chemical Toxicology.

[bb0025] Cao W., Wang Y., Zhuang Q., Wang L., Ni Y. (2019). Developing an electrochemical sensor for the detection of tert-butylhydroquinone. Sensors and Actuators B: Chemical.

[bb0035] Chen H., Liu S., Chen Y., Chen C., Yang H., Chen Y. (2020). Food safety management systems based on ISO 22000:2018 methodology of hazard analysis compared to ISO 22000:2005. Accreditation and Quality Assurance.

[bb0040] Chen X.F., Abdallah M., Chen X., Rajkovic A. (2023). Current knowledge of individual and combined toxicities of aflatoxin B1 and Fumonisin B1 in vitro. Toxins.

[bb0045] Chen Y., Waterhouse G.I.N., Sun H., Qiao X., Sun Y., Xu Z. (2022). Novel ratiometric electrochemical sensing platform with dual-functional poly-dopamine and NiS@HCS signal amplification for sunset yellow detection in foods. Food Chemistry.

[bb0050] Chen Z., Liu C., Su X., Zhang W., Zou X. (2021). Signal on-off ratiometric electrochemical sensor based on semi-complementary aptamer couple for sensitive cadmium detection in mussel. Sensors and Actuators B: Chemical.

[bb0055] Cui H., An K., Wang C., Chen Y., Jia S., Qian J., Hao N., Wei J., Wang K. (2022). A disposable ratiometric electrochemical aptasensor with exonuclease I-powered target recycling amplification for highly sensitive detection of aflatoxin B1. Sensors and Actuators B: Chemical.

[bb0060] Cui L., Li M., Zou X.R., Zhang C.Y. (2018). Advances in development of Ratiometric electrochemical sensors and their biochemical applications. Chinese Journal of Analytical Chemistry.

[bb0065] Deng X., Yi Z., Xiong Y., Gao X., Huang R., Chen X., Deng D., Xiong C., Zhang J., Huang G. (2024). Molecularly imprinted ratiometric electrochemical sensor based on 3D-1D MoS2@CNTs hetero-nanoflower for selective detection of trimethoprim. Microchemical Journal.

[bb0070] Diao E., Hou H., Dong H. (2013). Ozonolysis mechanism and influencing factors of aflatoxin B1: A review. Trends in Food Science & Technology.

[bb0075] Donno D., Mellano M.G., Gamba G., Riondato I., Beccaro G.L. (2020). Analytical Strategies for Fingerprinting of Antioxidants, Nutritional Substances, and Bioactive Compounds in Foodstuffs Based on High Performance Liquid Chromatography–Mass Spectrometry: An Overview. Foods.

[bb0080] Duan N., Wang H., Li Y., Yang S., Tian H., Sun B. (2021). The research progress of organic fluorescent probe applied in food and drinking water detection. Coordination Chemistry Reviews.

[bb0085] Duan S., Wu X., Gong Z., Wang J., Liu X., Wang Q., Wang Y., Dai H. (2024). Curcumin-based ratiometric electrochemical sensing interface for the detection of Cd^2+^ and Pb^2+^ in grain products. Colloids and Surfaces A: Physicochemical and Engineering Aspects.

[bb0090] Duan Z., Dong S., Dong Y., Gao Q. (2021). Geographical origin identification of two salmonid species via flavor compound analysis using headspace-gas chromatography-ion mobility spectrometry combined with electronic nose and tongue. Food Research International.

[bb0095] Gao X., Sun Z., Wang X., Zhang W., Wang Y., Han J., Sun X., Guo Y., Li F., Xu S. (2022). Construction of a ratiometric electrochemical aptasensor based on graphdiyne-methylene blue and fc-labeled hairpin for cyclic signal amplification detection of kanamycin. Sensors and Actuators B: Chemical.

[bb0100] Godfray H.C.J., Beddington J.R., Crute I.R., Haddad L., Lawrence D., Muir J.F., Toulmin C. (2010). Food security: The challenge of feeding 9 billion people. Science.

[bb0105] Gu Y., Li Y., Ren D., Sun L., Zhuang Y., Yi L., Wang S. (2022). Recent advances in nanomaterial-assisted electrochemical sensors for food safety analysis. Food Frontiers.

[bb0110] Guan Y., Si P.-B., Yang T., Wu Y., Yang Y.-H., Hu R. (2023). A novel method for detection of ochratoxin A in foods—Co-MOFs based dual signal ratiometric electrochemical aptamer sensor coupled with DNA walker. Food Chemistry.

[bb0115] Han K., Chen L., Zhang W., Tong Y., Shi J., Su X., Zou X. (2023). A ratiometric electrochemical sensor for detecting lead in fish based on the synergy of semi-complementary aptamer pairs and ag nanowires@zeolitic imidazolate framework-8. Analytical Methods.

[bb0120] Hou C.-Y., Fu L.-M., Ju W.-J., Wu P.-Y. (2020). Microfluidic colorimetric system for nitrite detection in foods. Chemical Engineering Journal.

[bb0125] Hu X., Liu Y., Xia Y., Zhao F., Zeng B. (2021). A novel ratiometric electrochemical sensor for the selective detection of citrinin based on molecularly imprinted poly(thionine) on ionic liquid decorated boron and nitrogen co-doped hierarchical porous carbon. Food Chemistry.

[bb0130] Hu X., Xia Y., Liu Y., Chen Y., Zeng B. (2022). An effective ratiometric electrochemical sensor for highly selective and reproducible detection of ochratoxin A: Use of magnetic field improved molecularly imprinted polymer. Sensors and Actuators B: Chemical.

[bb0135] Jia F., Li Y., Gong Q., Liu D., Meng S., Zhu C., You T. (2022). A Simple Ratiometric Electrochemical Aptasensor Based on the Thionine–Graphene Nanocomposite for Ultrasensitive Detection of Aflatoxin B2 in Peanut and Peanut Oil. Chemosensors.

[bb0140] Jiang Y., Sima Y., Liu L., Zhou C., Shi S., Wan K., Chen A., Tang N., He Q., Liu J. (2024). Research progress on portable electrochemical sensors for detection of mycotoxins in food and environmental samples. Chemical Engineering Journal.

[bb0145] Jin H., Sun Z., Sun Y., Gui R. (2021). Dual-signal ratiometric platforms: Construction principles and electrochemical biosensing applications at the live cell and small animal levels. TrAC Trends in Analytical Chemistry.

[bb0150] Jin Y., Zhang Y., Xu H., Lu X., Yuan Y., Zhang W. (2024). A ratiometric electrochemical aptasensor for sensitive detection of kanamycin in food based on entropy-driven strand displacement reaction. Food Control.

[bb0155] Kandemir K., Tomas M., McClements D.J., Capanoglu E. (2022). Recent advances on the improvement of quercetin bioavailability. Trends in Food Science & Technology.

[bb0160] Karuppaiah G., Koyappayil A., Go A., Lee M.-H. (2023). Ratiometric electrochemical detection of kojic acid based on glassy carbon modified MXene nanocomposite. RSC Advances.

[bb0165] Khezerlou A., Tavassoli M., Alizadeh Sani M., Ghasempour Z., Ehsani A., Khalilzadeh B. (2023). Rapid and sensitive detection of tetracycline residue in food samples using Cr(III)-MOF fluorescent sensor. Food Chemistry: X.

[bb0170] Kodr D., Yenice C.P., Simonova A., Saftić D.P., Pohl R., Sýkorová V., Hocek M. (2021). Carborane- or Metallacarborane-linked nucleotides for redox labeling. Orthogonal multipotential coding of all four DNA bases for electrochemical analysis and sequencing. Journal of the American Chemical Society.

[bb0175] Kumaragurubaran N., Arul P., Huang S.-T., Huang C.-H., Fang S.-B., Lin Y.-H. (2023). Nanocatalyst coupled with a latent-ratiometric electrochemical switch for label-free zero-tolerance rapid detection of live Salmonella in whole blood samples. Sensors and Actuators B: Chemical.

[bb0180] Kumaravel S., Jian S.-E., Huang S.-T., Huang C.-H., Hong W.-Z. (2022). Convenient and ultrasensitive detection of live Salmonella using ratiometric electrochemical molecular substrates. Analytica Chimica Acta.

[bb0185] Lai W.F., Wong W.T. (2022). Design and optimization of quercetin-based functional foods. Critical Reviews in Food Science and Nutrition.

[bb0190] Li H., Li Q., Zhao S., Wang X., Li F. (2022). Aptamer–Target Recognition-Promoted Ratiometric Electrochemical Strategy for Evaluating the Microcystin-LR Residue in Fish without Interferences. Journal of Agricultural and Food Chemistry.

[bb0195] Li J., Huang Y., Zhou Y., Dong H., Wang H., Shan H., Li Y., Xu M., Wang X. (2023). Controllable construction of two-dimensional conductive M_3_(HHTP)_2_ Nanorods for electrochemical sensing of malachite green in fish. ACS Applied Nano Materials.

[bb0200] Li J., Yang F., Chen X., Fang H., Zha C., Huang J., Liu Y. (2023). Dual-ratiometric aptasensor for simultaneous detection of malathion and profenofos based on hairpin tetrahedral DNA nanostructures. Biosensors and Bioelectronics.

[bb0205] Li Y.-L., Xie F.-T., Yao C., Zhang G.-Q., Guan Y., Yang Y.-H., Yang J.-M., Hu R. (2022). A DNA tetrahedral nanomaterial-based dual-signal ratiometric electrochemical aptasensor for the detection of ochratoxin A in corn kernel samples. Analyst.

[bb0210] Liang J., Zhang Y., Li Z., Lu X., Qi C., Yang Q., Zhang W. (2024). DNAzyme-driven tripedal DNA walker for ratiometric electrochemical aptasensor ultrasensitive detection of aflatoxin B1. Food Control.

[bb0215] Liang X., Zhao F., Xiao C., Yue S., Huang Y., Wei M. (2021). A ratiometric electrochemical aptasensor for ochratoxin A detection. Journal of the Chinese Chemical Society.

[bb0220] Lin Y., Wang J., Luo F., Guo L., Qiu B., Lin Z. (2018). Highly reproducible ratiometric aptasensor based on the ratio of amplified electrochemiluminescence signal and stable internal reference electrochemical signal. Electrochimica Acta.

[bb0225] Liu C., Wei X., Wang X., Shi J., Chen Z., Zhang H., Zhang W., Zou X. (2021). Ratiometric electrochemical analysis on a flexibly-fabricated vibratory electrode module for reliable and selective determination of imidacloprid. Sensors and Actuators B: Chemical.

[bb0230] Liu C., Wu T., Zeng W., Liu J., Hu B., Wu L. (2022). Dual-signal electrochemical aptasensor involving hybridization chain reaction amplification for aflatoxin B1 detection. Sensors and Actuators B: Chemical.

[bb0235] Liu D.-M., Dong C. (2023). Gold nanoparticles as colorimetric probes in food analysis: Progress and challenges. Food Chemistry.

[bb0240] Liu S., Meng S., Wang M., Li W., Dong N., Liu D., Li Y., You T. (2023). In-depth interpretation of aptamer-based sensing on electrode: Dual-mode electrochemical-photoelectrochemical sensor for the ratiometric detection of patulin. Food Chemistry.

[bb0245] Liu W., Dong H., Zhang L., Tian Y. (2017). Development of an efficient biosensor for the in vivo monitoring of cu^+^ and pH in the brain: Rational design and synthesis of recognition molecules. Angewandte Chemie International Edition.

[bb0250] Liu Y., Guo W., Zhang Y., Lu X., Yang Q., Zhang W. (2023). An accurate and ultrasensitive ratiometric electrochemical aptasensor for determination of Ochratoxin A based on catalytic hairpin assembly. Food Chemistry.

[bb0255] Luo S., Kan X. (2022). Specifically triggered dissociation based ratiometric electrochemical sensor for H_2_O_2_ measurement in food samples. Food Chemistry.

[bb0260] Luo Y., Lin R., Zuo Y., Zhang Z., Zhuo Y., Lu M., Chen S., Gu H. (2022). Efficient electrochemical microsensor for in vivo monitoring of H_2_O_2_ in PD mouse brain: Rational design and synthesis of recognition molecules. Analytical Chemistry.

[bb0265] Lv M., Li F., Du Y., Guo X., Zhang P., Liu Y. (2023). Ratiometric electrochemical aptasensor for AFB1 detection in peanut and peanut products. International Journal of Electrochemical Science.

[bb0270] Lv X., Xu X., Miao T., Zang X., Geng C., Li Y., Cui B., Fang Y. (2022). A ratiometric electrochemiluminescent/electrochemical strategy based on novel designed BPYHBF nanorod and fc-MOF with tungsten for ultrasensitive AFB1 detection. Sensors and Actuators B: Chemical.

[bb0275] Matsui Y., Tanaka Y., Iwahashi H. (2017). A comparative study of the inhibitory effects by caffeic acid, catechins and their related compounds on the generation of radicals in the reaction mixture of linoleic acid with iron ions. Journal of Clinical Biochemistry and Nutrition.

[bb0280] Meng S., Liu D., Li Y., Dong N., Chen T., You T. (2022). Engineering the signal transduction between CdTe and CdSe quantum dots for in situ Ratiometric Photoelectrochemical immunoassay of Cry1Ab protein. Journal of Agricultural and Food Chemistry.

[bb0285] Mohan B., Priyanka Singh G., Chauhan A., Pombeiro A.J.L., Ren P. (2023). Metal-organic frameworks (MOFs) based luminescent and electrochemical sensors for food contaminant detection. Journal of Hazardous Materials.

[bb0290] Moradi O. (2022). A review on nanomaterial-based electrochemical sensors for determination of vanillin in food samples. Food and Chemical Toxicology.

[bb0295] Naveed M., BiBi J., Kamboh A.A., Suheryani I., Kakar I., Fazlani S.A., XiaoHui Z. (2018). Pharmacological values and therapeutic properties of black tea (*Camellia sinensis*): A comprehensive overview. Biomedicine & Pharmacotherapy.

[bb0300] Neng J., Wang J., Wang Y., Zhang Y., Chen P. (2023). Trace analysis of food by surface-enhanced Raman spectroscopy combined with molecular imprinting technology: Principle, application, challenges, and prospects. Food Chemistry.

[bb0305] Niu X.H., Wang M.Z., Hu P.W., Liu B.X. (2022). A Ratiometric electroanalytical method based on diazotization reaction for detection of nitrite. Chinese Journal of Analytical Chemistry.

[bb0310] Nolvachai Y., Amaral M.S.S., Marriott P.J. (2023). Foods and contaminants analysis using multidimensional gas chromatography: An update of recent studies, technology, and applications. Analytical Chemistry.

[bb0315] Oh W.Y., Ambigaipalan P., Shahidi F. (2021). Quercetin and its ester derivatives inhibit oxidation of food, LDL and DNA. Food Chemistry.

[bb0320] Pan Y., Jiang J., Kan X. (2023). Diazo-reaction based dual-mode colorimetric-electrochemical sensing of nitrite in pickled food. Analyst.

[bb0325] Pang Y.-H., Wang Y.-Y., Shen X.-F., Qiao J.-Y. (2022). Covalent organic framework modified carbon cloth for ratiometric electrochemical sensing of bisphenol A and S. Microchimica Acta.

[bb0330] Peñalver R., Arroyo-Manzanares N., Campillo N., Viñas P. (2021). Targeted and untargeted gas chromatography-mass spectrometry analysis of honey samples for determination of migrants from plastic packages. Food Chemistry.

[bb0335] Qileng A., Huang S., He L., Qin W., Liu W., Xu Z., Liu Y. (2020). Composite films of CdS nanoparticles, MoS2 Nanoflakes, reduced graphene oxide, and carbon nanotubes for Ratiometric and modular Immunosensing-based detection of toxins in cereals. ACS Applied Nano Materials.

[bb0340] Qileng A., Liang H., Huang S., Liu W., Xu Z., Liu Y. (2020). Dual-function of ZnS/Ag2S nanocages in ratiometric immunosensors for the discriminant analysis of ochratoxins: Photoelectrochemistry and electrochemistry. Sensors and Actuators B: Chemical.

[bb0345] Qin Y., Liu S., Meng S., Liu D., You T. (2024). Split aptamer-based sandwich-type ratiometric biosensor for dual-modal photoelectrochemical and electrochemical detection of 17β-estradiol. Analytica Chimica Acta.

[bb0350] Riboni N., Bianchi F., Mattarozzi M., Caldara M., Gullì M., Graziano S., Maestri E., Marmiroli N., Careri M. (2023). Ultra-high Performance Liquid Chromatography–Ion Mobility–High-Resolution Mass Spectrometry to Evaluate the Metabolomic Response of Durum Wheat to Sustainable Treatments. Journal of Agricultural and Food Chemistry.

[bb0355] Sarlak Z., Khosravi-Darani K., Rouhi M., Garavand F., Mohammadi R., Sobhiyeh M.R. (2021). Bioremediation of organophosphorus pesticides in contaminated foodstuffs using probiotics. Food Control.

[bb0030] Schrenk D., Bodin L., Chipman J.K., del Mazo J., Grasl-Kraupp B., Bignami M. (2020). Risk assessment of ochratoxin A in food. EFSA Journal.

[bb0360] Shan X., Kuang D., Feng Q., Wu M., Yang J. (2023). A dual-mode ratiometric aptasensor for accurate detection of pathogenic bacteria based on recycling of DNAzyme activation. Food Chemistry.

[bb0365] Shan X., Xie H., Zhou T., Wu M., Yang J. (2023). Dual DNA recycling amplifications coupled with au NPs@ZIF-MOF accelerator for enhanced electrochemical ratiometric sensing of pathogenic bacteria. Talanta.

[bb0370] Shao W.J., Hou S.J., Jia W.K., Zheng Y.J. (2022). Rapid non-destructive analysis of food nutrient content using Swin-nutrition. Foods.

[bb0375] Shu Z., Hu H., Yuan Z., Zou Y., Zhang Q., Wang Y., Liu X., Duan S., Pi F., Wang J., Liu X., Dai H. (2024). Fe-MOF/AuNP-based ratiometric electrochemical immunosensor for the detection of deoxynivalenol in grain products. Microchimica Acta.

[bb0380] Similä M., Ovaskainen M.L., Virtanen M.J., Valsta L.M. (2006). Nutrient content patterns of Finnish foods in a food composition database. Journal of Food Composition and Analysis.

[bb0385] Singh H., Singh G., Kaur N., Singh N. (2022). Pattern-based colorimetric sensor array to monitor food spoilage using automated high-throughput analysis. Biosensors and Bioelectronics.

[bb0390] Spring S.A., Goggins S., Frost C.G. (2021). Ratiometric electrochemistry: Improving the robustness, reproducibility and reliability of biosensors. Molecules.

[bb0395] Su X., Chen Z., Wang H., Yuan L., Zheng K., Zhang W., Zou X. (2022). Ratiometric immunosensor with DNA tetrahedron nanostructure as high-performance carrier of reference signal and its applications in selective phoxim determination for vegetables. Food Chemistry.

[bb0400] Su X., Wang H., Wang C., Zhou X., Zou X., Zhang W. (2022). Programmable dual-electric-field immunosensor using MXene-au-based competitive signal probe for natural parathion-methyl detection. Biosensors and Bioelectronics.

[bb0405] Sun Y., Jiang X., Jin H., Gui R. (2019). Ketjen black/ferrocene dual-doped MOFs and aptamer-coupling gold nanoparticles used as a novel ratiometric electrochemical aptasensor for vanillin detection. Analytica Chimica Acta.

[bb0410] Sun Z., Xiao Q., Tang J., Zhuang Q., Wang Y. (2021). Ratiometric electrochemical sensor for bisphenol A detection using a glassy carbon electrode modified with a poly(toluidine blue)/gold nanoparticle composite. Analytical Methods.

[bb0415] Suo Z., Niu X., Liu R., Xin L., Liu Y., Wei M. (2022). A methylene blue and ag+ ratiometric electrochemical aptasensor based on au@Pt/Fe-N-C signal amplification strategy for zearalenone detection. Sensors and Actuators B: Chemical.

[bb0420] Tang J., Tao X. (2023). Gold nanoparticle-based aptasensors for detecting kanamycin in foods: Recent advances and perspectives. Microchemical Journal.

[bb0425] Tsiasioti A., Tzanavaras P.D. (2024). High performance liquid chromatography coupled with post – Column derivatization methods in food analysis: Chemistries and applications in the last two decades. Food Chemistry.

[bb0430] Tsiasioti A., Zotou A.-S., Tzanavaras P.D. (2021). Single run analysis of glutathione and its disulfide in food samples by liquid chromatography coupled to on-line post-column derivatization. Food Chemistry.

[bb0435] Wan C.-Q., Pang Y.-H., Feng Y.-W., Shen X.-F. (2022). A ratio fluorescence sensor based on rhodamine B embedded metal-organic framework for glyphosate detection in Agri-food products. Food Chemistry.

[bb0440] Wan Y.Q., Liu J.H., Pi F.W., Wang J.H. (2023). Advances on removal of organophosphorus pesticides with electrochemical technology. Critical Reviews in Food Science and Nutrition.

[bb0445] Wang C., Qian J., An K., Lu X., Huang X. (2019). A semiconductor quantum dot-based ratiometric electrochemical aptasensor for the selective and reliable determination of aflatoxin B1. Analyst.

[bb0450] Wang M., Zhao M., Liu P., Zhu H., Liu B., Hu P., Niu X. (2022). Coupling diazotization with oxidase-mimetic catalysis to realize dual-mode double-ratiometric colorimetric and electrochemical sensing of nitrite. Sensors and Actuators B: Chemical.

[bb0455] Wang S.-Y., Shi X.-C., Liu F.-Q., Laborda P. (2020). Chromatographic methods for detection and quantification of Carbendazim in food. Journal of Agricultural and Food Chemistry.

[bb0460] Wang W., Yin Y., Gunasekaran S. (2023). Nanozymatic degradation and simultaneous colorimetric detection of tetracycline. Food Chemistry.

[bb0465] Wang Y., Yu F., Liu Q., Wang C., Zhu G., Bai L., Shi S., Zhao Y., Jiang Z., Zhang W. (2024). A novel and sensitive dual signaling ratiometric electrochemical aptasensor based on nanoporous gold for determination of Ochratoxin A. Food Chemistry.

[bb0470] Wang Y., Zhai H., Guo Q., Zhang Y., Sun X., Guo Y., Yang Q., Zhang Y. (2023). Shared hairpin structure electrochemical aptasensor based on ZrO_2_@Ni/co-MOFs@AuNPs for dual-target detection of Cd^2+^ and S.Aureus. Sensors and Actuators B: Chemical.

[bb0475] Wei J., Liu C., Wu T., Zeng W., Hu B., Zhou S., Wu L. (2022). A review of current status of ratiometric molecularly imprinted electrochemical sensors: From design to applications. Analytica Chimica Acta.

[bb0480] Wen Y., Sun D., Zhang Y., Zhang Z., Chen L., Li J. (2023). Molecular imprinting-based ratiometric fluorescence sensors for environmental and food analysis. Analyst.

[bb0485] Wu L., Ding F., Yin W., Ma J., Wang B., Nie A., Han H. (2017). From electrochemistry to electroluminescence: Development and application in a Ratiometric Aptasensor for aflatoxin B1. Analytical Chemistry.

[bb0490] Wu L., Tang X., Wu T., Zeng W., Zhu X., Hu B., Zhang S. (2023). A review on current progress of Raman-based techniques in food safety: From normal Raman spectroscopy to SESORS. Food Research International.

[bb0495] Wu L., Wu T., Zeng W., Zhou S., Zhang W., Ma J. (2023). A new ratiometric molecularly imprinted electrochemical sensor for the detection of sunset yellow based on gold nanoparticles. Food Chemistry.

[bb0500] Xing L., Zhang W., Fu L., Lorenzo J.M., Hao Y. (2022). Fabrication and application of electrochemical sensor for analyzing hydrogen peroxide in food system and biological samples. Food Chemistry.

[bb0505] Xu M., Wang X., Liu X. (2022). Detection of heavy metal ions by Ratiometric photoelectric sensor. Journal of Agricultural and Food Chemistry.

[bb0510] Xu Q., Liu Z., Fu J., Zhao W., Guo Y., Sun X., Zhang H. (2017). Ratiometric electrochemical aptasensor based on ferrocene and carbon nanofibers for highly specific detection of tetracycline residues. Scientific Reports.

[bb0515] Xu T., Yang L., Zhang X., Lu G., Bai Z. (2023). A highly sensitive electrochemical sensor by growing ag nanoparticles on the surface of PPy@PEDOT:PSS film for detecting sodium hydroxymethanesulfinate molecules. Food Chemistry: X.

[bb0520] Xu Z., Li P., Liu X., Zhu X., Liu M., Zhang Y., Yao S. (2022). Dual-signal intrinsic self-calibration ratio electrochemical sensor for glutathione based on silver nanoparticle decorated Prussian blue analog. Electrochimica Acta.

[bb0525] Yang T., Yu R., Yan Y., Zeng H., Luo S., Liu N., Morrin A., Luo X., Li W. (2018). A review of ratiometric electrochemical sensors: From design schemes to future prospects. Sensors and Actuators B: Chemical.

[bb0530] Yin C., Wang Y., Zhuang Q. (2021). Dual-ratiometric electrochemical sensor for propyl gallate detection. Journal of Electroanalytical Chemistry.

[bb0535] Yin C., Zhuang Q., Xiao Q., Wang Y., Xie J. (2021). Electropolymerization of poly(methylene blue) on flower-like nickel-based MOFs used for ratiometric electrochemical sensing of total polyphenolic content in chrysanthemum tea. Analytical Methods.

[bb0540] Yin H., Zhang Y., Dong H., Liu L., Wang X., Zhang Y., Xu M., Zhou Y. (2022). Self-calibrating electrochemical sensors based on uniformly dispersed ag nanoclusters in nitrogen-doped carbon sheets for determination of nitrite. ACS Applied Nano Materials.

[bb0545] Yu H., Yuan N., Zhang Y., Guo W., Lu X., Yang Q., Zhang W. (2022). Saltatory rolling circle amplification-based Ratiometric electrochemical biosensor for rapid detection of Salmonella enterica serovar typhimurium in food. Food Analytical Methods.

[bb0550] Yu J., Jin H., Gui R., Lv W., Wang Z. (2017). A facile strategy for ratiometric electrochemical sensing of quercetin in electrolyte solution directly using bare glassy carbon electrode. Journal of Electroanalytical Chemistry.

[bb0555] Zeng W., Tang X., Wu T., Han B., Wu L. (2024). Development of a highly sensitive aptamer-based electrochemical sensor for detecting saxitoxin based on K3Fe(CN)6 regulated silver nanoparticles. Analytica Chimica Acta.

[bb0560] Zhang M., Zhang Z., Yang Y., Zhang Y., Wang Y., Chen X. (2020). Ratiometric strategy for electrochemical sensing of Carbaryl residue in water and vegetable samples. Sensors.

[bb0565] Zhang W., Liu C., Han K., Wei X., Xu Y., Zou X., Zhang H., Chen Z. (2020). A signal on-off ratiometric electrochemical sensor coupled with a molecular imprinted polymer for selective and stable determination of imidacloprid. Biosensors and Bioelectronics.

[bb0570] Zhang W., Ma J., Sun D.-W. (2021). Raman spectroscopic techniques for detecting structure and quality of frozen foods: Principles and applications. Critical Reviews in Food Science and Nutrition.

[bb0575] Zhang W., Wen J., Wang J., Yang K., Sun S. (2022). Recent development and application of ratiometric electrochemical biosensor. Journal of Electroanalytical Chemistry.

[bb0580] Zhang X., Li Z., Shi Y., Hu B., Zheng Q., Piao Y., Feng L., Cao J. (2024). Electrochemical/photoelectrochemical dual-mode aptasensor for sensitive aflatoxin B1 assay based on distance-modulation strategy using au NPs/PCZIF-8-ZnO as sensing substrate. Food Chemistry.

[bb0585] Zhang Y., Lei Y., Lu H., Shi L., Wang P., Ali Z., Li J. (2021). Electrochemical detection of bisphenols in food: A review. Food Chemistry.

[bb0590] Zhang Y., Liu X., Tian Y., Geng Y., Wang J., Ma M. (2024). A ratiometric electrochemical sensing strategy based on the self-assembly of co NC/CNT and methylene blue for effective detection of the food additive tert-butylhydroquinone. Talanta.

[bb0595] Zhang Z., Jia X., Xu X. (2023). An electrochemical aptasensor for detection of streptomycin based on signal amplification assisted by functionalized gold nanoparticles and hybridization chain reaction. Microchimica Acta.

[bb0600] Zhang Z., Li M., Zuo Y., Chen S., Zhuo Y., Lu M., Shi G., Gu H. (2022). In vivo monitoring of pH in subacute PD mouse brains with a Ratiometric electrochemical microsensor based on poly(melamine) films. ACS Sensors.

[bb0605] Zhao F., Guo D., Lan J., Liu Y. (2024). One-step electrodeposition of MWCNTs-cu MOF films for the ratiometric electrochemical analysis of glyphosate. Analytical Methods.

[bb0610] Zhao S.J., Wang J., He F., Du P.F., Liu T.D., Zhang S.Y., Li T.F. (2023). MB @ ZIF-8/COOH-MWCNTs composite-based Ratiometric electrochemical sensor for detection of hydrogen peroxide. Chinese Journal of Analytical Chemistry.

[bb0615] Zheng S., Yang Q., Yang H., Zhang Y., Guo W., Zhang W. (2023). An ultrasensitive and specific ratiometric electrochemical biosensor based on SRCA-CRISPR/Cas12a system for detection of Salmonella in food. Food Control.

[bb0620] Zhong W., Gao F., Zou J., Liu S., Li M., Gao Y., Yu Y., Wang X., Lu L. (2021). MXene@ag-based ratiometric electrochemical sensing strategy for effective detection of carbendazim in vegetable samples. Food Chemistry.

[bb0625] Zhou B., Xie H., Zhou S., Sheng X., Chen L., Zhong M. (2023). Construction of AuNPs/reduced graphene nanoribbons co-modified molecularly imprinted electrochemical sensor for the detection of zearalenone. Food Chemistry.

[bb0630] Zhou H., Qiu H., Zhang J., Fang Y., Cui B., Shen Y. (2024). Design, preparation, and application of molecularly imprinted nanomaterials for food safety analysis with electrochemistry. Coordination Chemistry Reviews.

[bb0635] Zhou T., Guo T., Wang Y., Wang A., Zhang M. (2023). Carbendazim: Ecological risks, toxicities, degradation pathways and potential risks to human health. Chemosphere.

[bb0640] Zhu C., Liu D., Li Y., Chen T., You T. (2022). Label-free ratiometric homogeneous electrochemical aptasensor based on hybridization chain reaction for facile and rapid detection of aflatoxin B1 in cereal crops. Food Chemistry.

[bb0645] Zhu C., Liu D., Li Y., Shen X., Ma S., Liu Y., You T. (2020). Ratiometric electrochemical aptasensor for ultrasensitive detection of Ochratoxin A based on a dual signal amplification strategy: Engineering the binding of methylene blue to DNA. Biosensors and Bioelectronics.

[bb0650] Zhu C., Wang X., Yang Y., Chen L., Yu D. (2023). Research progress on ratiometric electrochemical sensing of mycotoxins. Journal of Electroanalytical Chemistry.

